# Supplementary material for: Exposome-wide patterns predict brain health in aging
Source: Nat Commun. 2026 Apr 10;17:3409. doi: 10.1038/s41467-026-71271-9 (PMC13068930; doi:10.1038/s41467-026-71271-9)
Supplement: Supplementary file 1 — Supplementary Information [file 41467_2026_71271_MOESM1_ESM.pdf]

Supplementary Materials

Exposome-wide patterns predict brain health in aging

Mostafa Mahdipour[1,2], Somayeh Maleki Balajoo[1,2], Federico Raimondo[1,2], Jianxiao Wu[1,2], Eliana Nicolaisen-Sobesky[1,2], Shammi More[1,2,3], Felix Hoffstaedter[1,2], Holger Schwender[4], Masoud Tahmasian[1,2,5], Simon B. Eickhoff[1,2], and Sarah Genon[1,2,6]

[1] Institute of Neuroscience and Medicine (INM-7: Brain and Behaviour), Research Centre Jülich, Jülich, Germany
[2] Institute of Systems Neuroscience, Heinrich Heine University Düsseldorf, Düsseldorf, Germany
[3] Department of Bioinformatics, Fraunhofer Institute for Algorithms and Scientific Computing (SCAI), Sankt Augustin, Germany
[4] Mathematical Institute, Heinrich Heine University Düsseldorf, Düsseldorf, Germany

[5] Department of Nuclear Medicine, Faculty of Medicine and University Hospital Cologne, University of Cologne, Cologne, Germany

[6]GIGA-CRC-Human Imaging, University of Liège, Liège, Belgium.

Supplementary Table 1. Brain age prediction performance.

Supplementary Table 2. Full list of exposome variables.

Supplementary Table 3. Descriptive table for continuous variables in the Main subset.

Supplementary Table 4. Descriptive table for categorical variables in the Main subset.

Supplementary Table 5. Grey matter health prediction performances.

Supplementary Table 6. Feature importance ranking of all exposome variables in the main subset (*n* = 3706) based on different algorithms.

Supplementary Table 7. Feature importance ranking of all exposome variables in different subsets.

Supplementary Table 8. Summary statistics of coffee intake.

Supplementary Table 9. Summary of cereal type.

Supplementary Table 10. Summary statistics of hip circumference.

Supplementary Table 11. Correlation Between Chronological Age and Brain Age Gap (BAG) Before and After Bias Correction.

Supplementary Figure 1. SHAP-Based Contribution of Exposome Factors to Grey Matter Health Prediction in the Main Subset Using Linear SVR.

Supplementary Figure 2. SHAP-Based Contribution of Exposome Factors to Grey Matter Health Prediction in the Main Subset Using Ridge.

Supplementary Figure 3. SHAP-Based Contribution of Exposome Factors to Grey Matter Health Prediction in the Replication Subset Using Random Forest algorithm.

Supplementary Figure 4. SHAP-Based Contribution of Exposome Factors to Grey Matter Health Prediction in the Variables-Restricted Subset Using Random Forest algorithm.

Supplementary Figure 5. Similarity of Exposome factors' contribution in grey matter health prediction across different subsets/algorithms.

Supplementary Figure 6. Association Between Hip Circumference and Grey Matter Health in the Main Subset.

Supplementary Figure 7. Standardized Regression Coefficients for the Top Exposome Features Associated with Grey Matter Health in the Main Subset.

Supplementary Figure 8. Flowchart of Model Development for Brain Age and Grey Matter Health (Brain Age Gap) Predictions.

Supplementary Figure 9. Missingness pattern in the initial list of selected exposome variables.

Supplementary Figure 10. correlation heatmap of the preprocessed features.

Supplementary Figure 11. Distribution of exposome variables across different exposome domains.

Supplementary Figure 12. Fold-to-fold mean absolute SHAP values' correlations.

Supplementary Table 1. **Brain age prediction performance:** Brain age prediction performance using gray matter features across different levels of granularity and different predictive algorithms in held-out set of the healthy sample (*n* = 1005).

Model	Granularity level (cortical + subcortical parcellation)	Mean Absolute Error (MAE)	Correlation
Ridge Regression	**1000 + 54**	**3.73**	**0.76**
	800 + 54	3.77	0.76
	600 + 54	3.84	0.75
	400 + 54	3.85	0.75
	200 + 32	4.10	0.71
Linear Regression	1000 + 54	3.93	0.74
	800 + 54	3.86	0.74
	600 + 54	3.97	0.73
	400 + 54	3.90	0.74
	200 + 32	4.09	0.71
Support Vector Regression	1000 + 54	3.90	0.74
	800 + 54	3.88	0.74
	600 + 54	3.90	0.73
	400 + 54	4.02	0.71
	200 + 32	4.13	0.70
Random Forest	1000 + 54	4.56	0.64
	800 + 54	4.58	0.64
	600 + 54	5.73	0.44
	400 + 54	4.58	0.63
	200 + 32	4.63	0.63

Supplementary Table 2. **Full list of exposome variables:** Full list of exposome variables in three different subsets from UK Biobank including main subset (*n* = 3706, 261 exposome variables), replication subset (*n* = 4202, 259 exposome variables), and variable-restricted subset (*n* = 7736, 201 exposome variables).

#	Field ID	Instance	Title	Category	Main subset	Replication subset	variables-restricted subset
1	-1*	-1.0	Encoded anonymised participant ID	Basic sociodemographic	Yes	Yes	Yes
2	6138	0.0	Qualifications		Yes	Yes	Yes
3	-1*	-1.0	Years of education		Yes	Yes	Yes
4	-1*	-1.0	Race		Yes	Yes	Yes
5	1558	0.0	Alcohol intake frequency	Diet and alcohol	Yes	Yes	Yes
6	1369	0.0	Beef intake		Yes	Yes	Yes
7	1438	2.0	Bread intake		Yes	Yes	Yes
8	1448	0.0	Bread type		Yes	Yes	Yes
9	1458	0.0	Cereal intake		Yes	Yes	Yes
10	1408	0.0	Cheese intake		Yes	Yes	Yes
11	1498	0.0	Coffee intake		Yes	Yes	Yes
12	1289	0.0	Cooked vegetable intake		Yes	Yes	Yes
13	1319	0.0	Dried fruit intake		Yes	Yes	Yes
14	20411	0.0	Injury Caused by alcohol consumption		Yes	Yes	No
15	20405	0.0	Known person advocated alcohol reduction		Yes	Yes	No
16	20414	0.0	Alcohol intake		Yes	Yes	No
17	1309	0.0	Fresh fruit intake		Yes	Yes	Yes
18	1518	0.0	Hot drink temperature		Yes	Yes	Yes
19	1379	0.0	Lamb/mutton intake		Yes	Yes	Yes
20	1538	0.0	Major dietary changes in the last 5 years		Yes	Yes	Yes
21	1418	0.0	Milk type used		Yes	Yes	Yes
22	6144	2.0	Exclude eggs. dairy. wheat. sugar from diet		Yes	Yes	Yes
23	1339	0.0	Non-oily fish intake		Yes	Yes	Yes
24	1329	0.0	Oily fish intake		Yes	Yes	Yes
25	1389	0.0	Pork intake		Yes	Yes	Yes
26	1359	0.0	Poultry intake		Yes	Yes	Yes
27	1349	0.0	Processed meat intake		Yes	Yes	Yes
28	1299	0.0	Salad / raw vegetable intake		Yes	Yes	Yes
29	1478	0.0	Salt added to food		Yes	Yes	Yes
30	1428	2.0	Spread type		Yes	Yes	Yes
31	1488	0.0	Tea intake		Yes	Yes	Yes
32	1548	2.0	Variation in diet		Yes	Yes	Yes
33	6155	2.0	Vitamin and mineral supplements		Yes	Yes	Yes
34	1528	0.0	Water intake		Yes	Yes	Yes

35	894	2.0	Duration of moderate activity	Lifestyle and environment	Yes	Yes	Yes
36	1757	2.0	Facial ageing		Yes	Yes	Yes
37	20118	0.0	Home area population density - urban or rural		Yes	Yes	Yes
38	4836	2.0	Loud music exposure frequency		Yes	Yes	Yes
39	4825	2.0	Noisy workplace		Yes	Yes	Yes
40	20022	0.0	Birth weight	Early life factors	Yes	Yes	Yes
41	1687	0.0	Comparative body size at age 10		Yes	Yes	Yes
42	1697	0.0	Comparative height size at age 10		Yes	Yes	Yes
43	1707	0.0	Handedness (chirality/laterality)		Yes	Yes	Yes
44	1787	0.0	Maternal smoking around birth		Yes	Yes	Yes
45	3627	2.0	Angina : diagnostic age	Cardiovascular health	Yes	Yes	Yes
46	3894	2.0	Heart attack : diagnostic age		Yes	Yes	Yes
47	2966	2.0	High blood pressure : diagnostic age		Yes	Yes	Yes
48	4022	2.0	Pulmonary embolism (blood clot in lung) : diagnostic age		Yes	Yes	Yes
49	3436	0.0	Smoking : age when started again (current smokers)		Yes	Yes	Yes
50	2867	2.0	Smoking : age when started		Yes	Yes	Yes
51	2897	2.0	Smoking : age when stopped		Yes	Yes	Yes
52	4056	2.0	Stroke : diagnostic age		Yes	Yes	Yes
53	20455	0.0	Cannabis : age when last took		Yes	Yes	Yes
54	6152	0.0	Blood Clot. DVT. and Respiratory Conditions		Yes	Yes	Yes
55	4079	0.0	Diastolic blood pressure. automated reading		Yes	Yes	Yes
56	-1**	-1.0	Smoking : duration		Yes	Yes	Yes
57	3627	2.0	Angina : diagnostic duration		Yes	Yes	Yes
58	3894	2.0	Heart attack : diagnostic duration		Yes	Yes	Yes
59	2966	2.0	High blood pressure : diagnostic duration		Yes	Yes	Yes
60	4022	2.0	Pulmonary embolism (blood clot in lung) : diagnostic duration		Yes	Yes	Yes
61	4056	2.0	Stroke : diagnostic duration		Yes	Yes	Yes
62	20455	0.0	Cannabis : duration from when last took		Yes	Yes	Yes
63	20453	0.0	Cannabis usage		Yes	Yes	No
64	4194	2.0	Pulse rate		Yes	Yes	Yes
65	102	0.0	Pulse rate. automated reading		Yes	Yes	Yes
66	21021	2.0	Pulse wave Arterial Stiffness index		Yes	Yes	Yes
67	20116	0.0	Smoking status		Yes	Yes	Yes
68	4080	0.0	Systolic blood pressure. automated reading		Yes	Yes	Yes
69	6150	0.0	Vascular/heart problems		Yes	Yes	Yes
70	4106	2.0	Heel bone mineral density (BMD) T-score. automated (left)	Bone health	Yes	No	No
71	4125	2.0	Heel bone mineral density (BMD) T-score. automated (right)		Yes	No	No

72	2976	2.0	Diabetes disease : diagnostic age	Diabetes	Yes	Yes	Yes
73	5901	2.0	diabetes-related eye disease : diagnostic age		Yes	Yes	Yes
74	2443	0.0	Diabetes : diagnosis		Yes	Yes	Yes
75	2976	2.0	Diabetes disease : diagnostic duration		Yes	Yes	Yes
76	5901	2.0	Diabetes-related eye disease : diagnostic duration		Yes	Yes	Yes
77	4700	2.0	Cataract : diagnostic age	Eye/Vision impairment	Yes	Yes	Yes
78	4689	2.0	Glaucoma : diagnostic age		Yes	Yes	Yes
79	5923	2.0	Macular degeneration : diagnostic age		Yes	Yes	Yes
80	2217	2.0	Wearing glasses or lenses : age when started		Yes	Yes	Yes
81	5430	2.0	Loss of vision due to injury or trauma: diagnostic age		Yes	Yes	Yes
82	4700	2.0	Cataract : diagnostic duration		Yes	Yes	Yes
83	4689	2.0	Glaucoma : diagnostic duration		Yes	Yes	Yes
84	5923	2.0	Macular degeneration : diagnostic duration		Yes	Yes	Yes
85	2217	2.0	Wearing glasses or lenses : duration from when started		Yes	Yes	Yes
86	5430	2.0	Loss of vision due to injury or trauma: diagnostic duration		Yes	Yes	Yes
87	22150	0.0	Chronic Obstructive Pulmonary Disease : diagnostic age	Body injuries and illnesses	Yes	Yes	Yes
88	3786	2.0	Asthma : diagnostic age		Yes	Yes	Yes
89	22147	0.0	Asthma : diagnostic age by doctor		Yes	Yes	Yes
90	22149	0.0	Chronic bronchitis : diagnostic age		Yes	Yes	Yes
91	22148	0.0	Emphysema : diagnostic age		Yes	Yes	Yes
92	3992	2.0	Emphysema/Chronic bronchitis : diagnostic age		Yes	Yes	Yes
93	3761	2.0	Hay fever. rhinitis or eczema : diagnostic age		Yes	Yes	Yes
94	22146	0.0	Hayfever or allergic rhinitis : diagnostic age		Yes	Yes	Yes
95	22160	0.0	Lung cancer (not mesothelioma) : diagnostic age		Yes	Yes	Yes
96	22153	0.0	Sarcoidosis : diagnostic age		Yes	Yes	Yes
97	22157	0.0	Tuberculosis : diagnostic age		Yes	Yes	Yes
98	2453	0.0	Cancer : diagnosis		Yes	Yes	Yes
99	2335	0.0	Chest pain or discomfort		Yes	Yes	Yes
100	20528	0.0	Life-threatening illness		Yes	Yes	No
101	22150	0.0	Chronic Obstructive Pulmonary Disease : diagnostic duration		Yes	Yes	Yes
102	3786	2.0	Asthma : diagnostic duration		Yes	Yes	Yes
103	22147	0.0	Asthma : diagnostic (by doctor) duration		Yes	Yes	Yes
104	22149	0.0	Chronic bronchitis : diagnostic age		Yes	Yes	Yes
105	22148	0.0	Emphysema : diagnostic duration		Yes	Yes	Yes
106	3992	2.0	Emphysema/Chronic bronchitis : diagnostic duration		Yes	Yes	Yes
107	3761	2.0	Hay fever. rhinitis or eczema : diagnostic duration		Yes	Yes	Yes
108	22146	0.0	Hayfever or allergic rhinitis : diagnostic duration		Yes	Yes	Yes

109	22160	0.0	Lung cancer (not mesothelioma) : diagnostic duration		Yes	Yes	Yes
110	22153	0.0	Sarcoidosis : diagnostic duration		Yes	Yes	Yes
111	22157	0.0	Tuberculosis : diagnostic duration		Yes	Yes	Yes
112	20007	2.0	Cancer : diagnostic duration (first time)		Yes	Yes	Yes
113	20007	02.01	Cancer : diagnostic duration (second time)		Yes	Yes	Yes
114	20009	2.0	Non-cancer illness: diagnostic duration (first time)		Yes	Yes	Yes
115	20009	02.01	Non-cancer illness: diagnostic duration (second time)		Yes	Yes	Yes
116	20009	02.02	Non-cancer illness: diagnostic duration (third time)		Yes	Yes	Yes
117	20009	02.03	Non-cancer illness: diagnostic duration (fourth time)		Yes	Yes	Yes
118	20009	02.04	Non-cancer illness: diagnostic duration (fifth time)		Yes	Yes	Yes
119	20009	02.05	Non-cancer illness: diagnostic duration (sixth time)		Yes	Yes	Yes
120	20009	02.06	Non-cancer illness: diagnostic duration (seventh time)		Yes	Yes	Yes
121	20009	02.07	Non-cancer illness: diagnostic duration (eighth time)		Yes	Yes	Yes
122	20011	2.0	Operation : duration of taking place (first time)		Yes	Yes	Yes
123	20011	02.01	Operation : duration of taking place (second time)		Yes	Yes	Yes
124	20011	02.02	Operation : duration of taking place (third time)		Yes	Yes	Yes
125	20011	02.03	Operation : duration of taking place (fourth time)		Yes	Yes	Yes
126	20011	02.04	Operation : duration of taking place (fifth time)		Yes	Yes	Yes
127	20011	02.05	Operation : duration of taking place (sixth time)		Yes	Yes	Yes
128	20011	02.06	Operation : age when took place (sixth time)		Yes	Yes	Yes
129	2296	0.0	Falls in the last year		Yes	Yes	Yes
130	20007	2.0	Cancer : diagnostic age (first time)		Yes	Yes	Yes
131	20007	02.01	Cancer : diagnostic age (second time)		Yes	Yes	Yes
132	20009	2.0	Non-cancer illness: diagnostic age (first time)		Yes	Yes	Yes
133	20009	02.01	Non-cancer illness: diagnostic age (second time)		Yes	Yes	Yes
134	20009	02.02	Non-cancer illness: diagnostic age (third time)		Yes	Yes	Yes
135	20009	02.03	Non-cancer illness: diagnostic age (fourth time)		Yes	Yes	Yes
136	20009	02.04	Non-cancer illness: diagnostic age (fifth time)		Yes	Yes	Yes
137	20009	02.05	Non-cancer illness: diagnostic age (sixth time)		Yes	Yes	Yes
138	20009	02.06	Non-cancer illness: diagnostic age (seventh time)		Yes	Yes	Yes
139	20009	02.07	Non-cancer illness: diagnostic age (eighth time)		Yes	Yes	Yes
140	20011	2.0	Operation : age when took place (first time)		Yes	Yes	Yes
141	20011	02.01	Operation : age when took place (second time)		Yes	Yes	Yes
142	20011	02.02	Operation : age when took place (third time)		Yes	Yes	Yes
143	20011	02.03	Operation : age when took place (fourth time)		Yes	Yes	Yes
144	20011	02.04	Operation : age when took place (fifth time)		Yes	Yes	Yes
145	2188	0.0	Long-standing illness. disability or infirmity		Yes	Yes	Yes
146	6154	2.0	Medication for pain relief. constipation. heartburn		Yes	Yes	Yes
147	6179	2.0	Mineral and other dietary supplements		Yes	Yes	Yes
148	136	0.0	Operations count : self-reported number		Yes	Yes	Yes

149	134	0.0	Cancers count : self-reported number		Yes	Yes	Yes
150	135	0.0	Non-cancer illnesses : self-reported number		Yes	Yes	Yes
151	2473	0.0	Other serious medical conditions and disabilities		Yes	Yes	Yes
152	2178	0.0	Overall health rating		Yes	Yes	Yes
153	6159	0.0	Pain type(s) experienced in last month		Yes	Yes	Yes
154	2492	0.0	Other prescription medications		Yes	Yes	Yes
155	2316	0.0	Wheeze or whistling in the chest in last year		Yes	Yes	Yes
156	26202	0.0	Standard PRS for age at menopause (AAM)	Aging susceptibility internal factors	Yes	Yes	Yes
157	26204	0.0	Standard PRS for age-related macular degeneration (AMD)		Yes	Yes	Yes
158	23124	0.0	Arm fat mass (left)	Body morphology	Yes	Yes	Yes
159	23120	0.0	Arm fat mass (right)		Yes	Yes	Yes
160	23123	0.0	Arm fat percentage (left)		Yes	Yes	Yes
161	23119	0.0	Arm fat percentage (right)		Yes	Yes	Yes
162	23125	0.0	Arm fat-free mass (left)		Yes	Yes	Yes
163	23121	0.0	Arm fat-free mass (right)		Yes	Yes	Yes
164	23126	0.0	Arm predicted mass (left)		Yes	Yes	Yes
165	23122	0.0	Arm predicted mass (right)		Yes	Yes	Yes
166	23105	0.0	Basal metabolic rate		Yes	Yes	Yes
167	23099	0.0	Body fat percentage		Yes	Yes	Yes
168	21001	0.0	Body mass index (BMI)		Yes	Yes	Yes
169	3063	0.0	Forced expiratory volume in 1-second (FEV1)		Yes	Yes	Yes
170	3062	0.0	Forced vital capacity (FVC)		Yes	Yes	Yes
171	46	0.0	Hand grip strength (left)		Yes	Yes	Yes
172	47	0.0	Hand grip strength (right)		Yes	Yes	Yes
173	49	0.0	Hip circumference		Yes	Yes	Yes
174	23110	0.0	Impedance of arm (left)		Yes	Yes	Yes
175	23109	0.0	Impedance of arm (right)		Yes	Yes	Yes
176	23108	0.0	Impedance of leg (left)		Yes	Yes	Yes
177	23107	0.0	Impedance of leg (right)		Yes	Yes	Yes
178	23106	0.0	Impedance of whole body		Yes	Yes	Yes
179	23116	0.0	Leg fat mass (left)		Yes	Yes	Yes
180	23112	0.0	Leg fat mass (right)		Yes	Yes	Yes
181	23115	0.0	Leg fat percentage (left)		Yes	Yes	Yes
182	23111	0.0	Leg fat percentage (right)		Yes	Yes	Yes
183	23117	0.0	Leg fat-free mass (left)		Yes	Yes	Yes
184	23113	0.0	Leg fat-free mass (right)		Yes	Yes	Yes
185	23118	0.0	Leg predicted mass (left)		Yes	Yes	Yes

186	23114	0.0	Leg predicted mass (right)		Yes	Yes	Yes
187	3064	0.0	Peak expiratory flow (PEF)		Yes	Yes	Yes
188	20015	0.0	Sitting height		Yes	Yes	Yes
189	23128	0.0	Trunk fat mass		Yes	Yes	Yes
190	23127	0.0	Trunk fat percentage		Yes	Yes	Yes
191	23129	0.0	Trunk fat-free mass		Yes	Yes	Yes
192	23130	0.0	Trunk predicted mass		Yes	Yes	Yes
193	48	0.0	Waist circumference		Yes	Yes	Yes
194	21002	0.0	Weight		Yes	Yes	Yes
195	2306	0.0	Weight change in last year		Yes	Yes	Yes
196	23100	0.0	Whole body fat mass		Yes	Yes	Yes
197	23101	0.0	Whole body fat-free mass		Yes	Yes	Yes
198	23102	0.0	Whole body water mass		Yes	Yes	Yes
199	20525	0.0	Able to pay rent/mortgage as an adult	Mental health	Yes	Yes	Yes
200	20433	0.0	Depression : age at first episode		Yes	Yes	Yes
201	20434	0.0	Depression : age at last episode		Yes	Yes	Yes
202	20461	0.0	Unusual or psychotic experience : diagnostic age		Yes	Yes	Yes
203	20495	0.0	Activities Avoided Due to Recent Stress		Yes	Yes	No
204	20522	0.0	Confiding Relationships in Adulthood		Yes	Yes	No
205	20526	0.0	Serious Life-Threatening Accident		Yes	Yes	No
206	20527	0.0	Combat/War-Zone Involvement		Yes	Yes	No
207	20460	0.0	Belief that own life is meaningful		Yes	Yes	No
208	20521	0.0	Belittlement by partner or ex-partner as an adult		Yes	Yes	No
209	20433	0.0	Depression : duration from first episode		Yes	Yes	Yes
210	20434	0.0	Depression : duration from last episode		Yes	Yes	Yes
211	20461	0.0	Unusual or psychotic experience : diagnostic duration		Yes	Yes	Yes
212	20401	0.0	Addiction to any substance or behaviour		Yes	Yes	No
213	20468	0.0	Belief in a personal conspiracy		Yes	Yes	No
214	20474	0.0	Belief in unreal communications		Yes	Yes	No
215	20485	0.0	Contemplated self-harm		Yes	Yes	No
216	20421	0.0	Long-term worry and tension		Yes	Yes	No
217	20502	0.0	Period of extreme irritability		Yes	Yes	No
218	20501	0.0	Period of mania / excitability		Yes	Yes	No
219	20446	0.0	Long-lasting sadness or depression		Yes	Yes	No
220	20441	0.0	Long-term loss of interest in activities		Yes	Yes	No
221	20463	0.0	heard an un-real voice		Yes	Yes	No
222	20471	0.0	seen an un-real vision		Yes	Yes	No
223	20480	0.0	Ever self-harmed		Yes	Yes	No
224	20499	0.0	Sought help for mental distress		Yes	Yes	No
225	20500	0.0	Mental distress affecting daily activities		Yes	Yes	No

226	20479	0.0	Thought life was not worth living		Yes	Yes	No
227	20425	0.0	Worried more than others in similar situations		Yes	Yes	No
228	4559	2.0	Family relationship satisfaction		Yes	Yes	Yes
229	20487	0.0	Felt hated by family member as a child		Yes	Yes	No
230	20489	0.0	Felt loved as a child		Yes	Yes	No
231	20498	0.0	Felt upset by reminders of past stress		Yes	Yes	No
232	4581	2.0	Financial situation satisfaction		Yes	Yes	Yes
233	4570	2.0	Friendships satisfaction		Yes	Yes	Yes
234	20458	0.0	General happiness		Yes	Yes	No
235	20459	0.0	General happiness with own health		Yes	Yes	No
236	20523	0.0	Physical violence from ex-/partner		Yes	Yes	No
237	20488	0.0	Physically abused by family as a child		Yes	Yes	No
238	20518	0.0	Changes in speed/amount of moving or speaking lately		Yes	Yes	No
239	20505	0.0	Easily annoyed or irritable lately		Yes	Yes	No
240	20510	0.0	Feelings of depression lately		Yes	Yes	No
241	20512	0.0	Feelings of foreboding lately		Yes	Yes	No
242	20507	0.0	Feelings of inadequacy lately		Yes	Yes	No
243	20519	0.0	Feelings of tiredness/low energy lately		Yes	Yes	No
244	20506	0.0	Feelings of anxiety/nervousness lately		Yes	Yes	No
245	20509	0.0	Difficulty stopping worry lately		Yes	Yes	No
246	20514	0.0	Diminished pleasure in activities lately		Yes	Yes	No
247	20511	0.0	Poor appetite and overeating lately		Yes	Yes	No
248	20516	0.0	Feelings of restlessness lately		Yes	Yes	No
249	20513	0.0	Having thoughts of self-harm lately		Yes	Yes	No
250	20508	0.0	Trouble focusing on tasks lately		Yes	Yes	No
251	20515	0.0	Difficulty relaxing lately		Yes	Yes	No
252	20520	0.0	Excessive worrying lately		Yes	Yes	No
253	20497	0.0	Disturbing thoughts of past stress lately		Yes	Yes	No
254	20524	0.0	Non-consensual sexual Interference by ex-/partners		Yes	Yes	No
255	20490	0.0	Sexually molested as a child		Yes	Yes	No
256	20491	0.0	Childhood Health: The Role of a Supportive Caregiver		Yes	Yes	No
257	20517	0.0	Sleep Disruptions: Difficulty Falling or Staying Asleep		Yes	Yes	No
258	20529	0.0	Victim of physically violent crime		Yes	Yes	No
259	20531	0.0	Victim of sexual assault		Yes	Yes	No
260	20530	0.0	Witnessed sudden violent death		Yes	Yes	No
261	4537	2.0	Work/job satisfaction		Yes	Yes	Yes

*: These exposomes has been converted by ukbb_parser package (https://github.com/USC-IGC/ukbb_parser)

- Race (Participant Race): Converted from and uses same data encoding as Data-Field 21000 which its encoding is 1001
- YearsOfEducation (Estimated Years of Education): Converted from Data-Field 6138 Qualifications which its encoding is 100305
- ISCED (Encoded anonymised participant ID): Converted from Data-Field 6138 Qualifications which its encoding is 100305

**: This exposome has been calculated by ourselves based on these Data-Fields:

- 2867: Age started smoking in former smokers
- 2897: Age stopped smoking
- 3436: Age started smoking in current smokers
- 6194: Age stopped smoking cigarettes (current cigar/pipe or previous cigarette smoker)

Supplementary Table 3. Descriptive table for continuous variables in the Main subset.

	Feature	Type	Mean ± SD	Median [IQR]	Min – Max	coding ID
0	Diabetes disease : diagnostic duration	Continuous	0.41 ± 2.99	0.00 [0.00, -0.00]	-0.00 – 59.00	100291.0
1	Impedance of whole body	Continuous	606.95 ± 86.77	604.00 [540.00, 667.00]	52.00 – 946.00	0.0
2	Impedance of leg (right)	Continuous	249.48 ± 33.29	248.00 [226.00, 272.00]	107.00 – 371.00	0.0
3	Non-cancer illness: diagnostic age (second time)	Continuous	23.14 ± 26.22	6.50 [0.00, 50.50]	0.00 – 77.80	13.0
4	Weight	Continuous	76.31 ± 14.65	74.80 [65.20, 85.38]	38.70 – 175.20	0.0
5	Glaucoma : diagnostic age	Continuous	1.21 ± 8.42	0.00 [0.00, 0.00]	0.00 – 76.00	100291.0
6	Non-cancer illness: diagnostic duration (seventh time)	Continuous	2.68 ± 10.89	0.00 [0.00, -0.00]	-0.00 – 71.50	13.0
7	Cancer : diagnostic age (second time)	Continuous	0.61 ± 6.10	0.00 [0.00, 0.00]	0.00 – 77.50	13.0
8	Hand grip strength (right)	Continuous	34.38 ± 11.02	32.00 [26.00, 42.00]	4.00 – 82.00	0.0
9	Whole body fat mass	Continuous	22.98 ± 8.33	21.80 [17.30, 27.20]	5.00 – 93.10	0.0
10	Body mass index (BMI)	Continuous	26.33 ± 4.09	25.75 [23.54, 28.42]	15.70 – 63.58	0.0
11	Cooked vegetable intake	Continuous	2.69 ± 1.64	2.00 [2.00, 3.00]	0.00 – 43.00	100373.0
12	Tuberculosis : diagnostic duration	Continuous	0.09 ± 2.19	0.00 [0.00, -0.00]	-0.00 – 63.00	0.0
13	Waist circumference	Continuous	87.02 ± 12.43	86.00 [77.00, 95.00]	55.00 – 142.00	0.0
14	Cannabis : duration from when last took	Continuous	6.54 ± 13.58	0.00 [0.00, -0.00]	-0.00 – 59.00	0.0
15	Smoking : age when started again (current smokers)	Continuous	0.60 ± 3.36	0.00 [0.00, 0.00]	0.00 – 50.00	100291.0
16	Asthma : diagnostic (by doctor) duration	Continuous	2.85 ± 10.48	0.00 [0.00, -0.00]	-0.00 – 74.00	0.0
17	Pulse rate	Continuous	70.27 ± 12.24	69.00 [62.00, 78.00]	36.00 – 160.00	0.0
18	Cereal intake	Continuous	4.85 ± 2.61	6.00 [3.00, 7.00]	0.00 – 15.00	100373.0
19	Asthma : diagnostic duration	Continuous	3.58 ± 11.92	0.00 [0.00, -0.00]	-0.00 – 78.00	100291.0
20	Operation : duration of taking place (third time)	Continuous	7.03 ± 14.81	0.00 [0.00, 3.50]	-0.00 – 73.50	13.0
21	Diabetes disease : diagnostic age	Continuous	2.07 ± 10.63	0.00 [0.00, 0.00]	0.00 – 79.00	100291.0
22	Leg fat mass (right)	Continuous	3.99 ± 1.65	3.80 [2.80, 4.80]	0.80 – 26.50	0.0
23	Non-cancer illnesses : self-reported number	Continuous	1.45 ± 1.60	1.00 [0.00, 2.00]	0.00 – 17.00	0.0
24	Cataract : diagnostic age	Continuous	6.35 ± 19.11	0.00 [0.00, 0.00]	0.00 – 78.00	100291.0
25	Arm fat mass (left)	Continuous	1.19 ± 0.59	1.10 [0.80, 1.40]	0.20 – 12.30	0.0
26	Basal metabolic rate	Continuous	6609.38 ± 1327.50	6305.00 [5489.00, 7622.00]	4079.00 – 11527.00	0.0
27	Non-cancer illness: diagnostic duration (second time)	Continuous	10.18 ± 16.68	0.30 [0.00, 13.50]	-0.00 – 77.50	13.0
28	Loss of vision due to injury or trauma: diagnostic duration	Continuous	0.13 ± 2.47	0.00 [0.00, -0.00]	-0.00 – 64.00	100291.0
29	Hip circumference	Continuous	101.99 ± 7.99	101.00 [97.00, 106.00]	77.00 – 168.00	0.0
30	Hayfever or allergic rhinitis : diagnostic age	Continuous	4.68 ± 12.32	0.00 [0.00, 0.00]	0.00 – 70.00	0.0

**31**	Chronic Obstructive Pulmonary Disease : diagnostic duration	Continuous	0.03 ± 0.89	0.00 [0.00, -0.00]	-0.00 – 49.00	0.0
**32**	Hay fever, rhinitis or eczema : diagnostic duration	Continuous	9.20 ± 18.15	0.00 [0.00, -0.00]	-0.00 – 77.00	100291.0
**33**	Water intake	Continuous	2.60 ± 2.15	2.00 [1.00, 4.00]	0.00 – 25.00	100373.0
**34**	Macular degeneration : diagnostic age	Continuous	0.87 ± 7.25	0.00 [0.00, 0.00]	0.00 – 78.00	100291.0
**35**	Non-cancer illness: diagnostic age (first time)	Continuous	34.66 ± 25.01	42.50 [4.50, 55.70]	0.00 – 78.20	13.0
**36**	Pulmonary embolism (blood clot in lung) : diagnostic duration	Continuous	0.07 ± 1.23	0.00 [0.00, -0.00]	-0.00 – 35.00	100291.0
**37**	Smoking : age when stopped	Continuous	7.81 ± 16.14	0.00 [0.00, 0.00]	0.00 – 73.00	100291.0
**38**	Non-cancer illness: diagnostic age (sixth time)	Continuous	4.15 ± 13.77	0.00 [0.00, 0.00]	0.00 – 73.90	13.0
**39**	Lung cancer (not mesothelioma) : diagnostic duration	Continuous	0.00 ± 0.28	0.00 [0.00, -0.00]	-0.00 – 17.00	0.0
**40**	Leg fat-free mass (left)	Continuous	8.87 ± 1.94	8.40 [7.20, 10.40]	5.30 – 16.90	0.0
**41**	Arm fat-free mass (left)	Continuous	2.91 ± 0.83	2.70 [2.20, 3.60]	1.50 – 5.70	0.0
**42**	Diabetes-related eye disease : diagnostic duration	Continuous	0.03 ± 0.57	0.00 [0.00, -0.00]	-0.00 – 18.00	100291.0
**43**	Macular degeneration : diagnostic duration	Continuous	0.10 ± 1.21	0.00 [0.00, -0.00]	-0.00 – 34.00	100291.0
**44**	Standard PRS for age at menopause (AAM)	Continuous	-0.09 ± 1.01	-0.10 [-0.77, 0.58]	-3.39 – 3.46	0.0
**45**	Standard PRS for age-related macular degeneration (AMD)	Continuous	0.10 ± 1.02	0.08 [-0.62, 0.81]	-3.24 – 3.69	0.0
**46**	Cannabis : age when last took	Continuous	7.48 ± 14.87	0.00 [0.00, 0.00]	0.00 – 74.00	0.0
**47**	Operation : duration of taking place (fourth time)	Continuous	3.25 ± 10.61	0.00 [0.00, -0.00]	-0.00 – 71.50	13.0
**48**	Heel bone mineral density (BMD) T-score, automated (right)	Continuous	-0.24 ± 1.19	-0.31 [-1.01, 0.43]	-5.49 – 7.99	0.0
**49**	Non-cancer illness: diagnostic duration (third time)	Continuous	8.02 ± 16.15	0.00 [0.00, 6.48]	-0.00 – 74.50	13.0
**50**	Arm fat mass (right)	Continuous	1.12 ± 0.53	1.00 [0.80, 1.30]	0.20 – 10.70	0.0
**51**	Operations count : self-reported number	Continuous	1.52 ± 1.37	1.00 [1.00, 2.00]	0.00 – 10.00	0.0
**52**	Coffee intake	Continuous	2.02 ± 1.96	2.00 [0.50, 3.00]	0.00 – 20.00	100373.0
**53**	Stroke : diagnostic duration	Continuous	0.08 ± 0.99	0.00 [0.00, -0.00]	-0.00 – 28.00	100291.0
**54**	Angina : diagnostic duration	Continuous	0.17 ± 1.66	0.00 [0.00, -0.00]	-0.00 – 36.00	100291.0
**55**	Hand grip strength (left)	Continuous	32.15 ± 10.91	30.00 [24.00, 40.00]	2.00 – 72.00	0.0
**56**	High blood pressure : diagnostic duration	Continuous	2.43 ± 6.35	0.00 [0.00, -0.00]	-0.00 – 51.00	100291.0
**57**	Leg fat percentage (right)	Continuous	30.58 ± 10.12	32.00 [21.30, 39.00]	7.80 – 61.50	0.0
**58**	Leg predicted mass (right)	Continuous	8.51 ± 1.87	8.10 [6.90, 10.00]	5.10 – 15.70	0.0
**59**	Sitting height	Continuous	90.09 ± 4.71	90.00 [87.00, 93.00]	71.00 – 117.00	0.0
**60**	Pulse rate, automated reading	Continuous	67.19 ± 10.55	67.00 [60.00, 73.00]	32.00 – 125.00	0.0
**61**	Leg predicted mass (left)	Continuous	8.40 ± 1.85	8.00 [6.80, 9.88]	5.00 – 15.90	0.0
**62**	Emphysema/Chronic bronchitis : diagnostic age	Continuous	0.33 ± 4.39	0.00 [0.00, 0.00]	0.00 – 73.00	100291.0
**63**	Trunk fat-free mass	Continuous	29.70 ± 5.92	28.20 [24.60, 34.60]	15.40 – 48.80	0.0
**64**	Arm fat percentage (left)	Continuous	28.79 ± 9.29	27.60 [21.30, 35.30]	8.90 – 73.00	0.0

**65**	High blood pressure : diagnostic age	Continuous	11.03 ± 21.98	0.00 [0.00, 0.00]	0.00 – 78.00	100291.0
**66**	Pulmonary embolism (blood clot in lung) : diagnostic age	Continuous	0.30 ± 3.93	0.00 [0.00, 0.00]	0.00 – 73.00	100291.0
**67**	Tuberculosis : diagnostic age	Continuous	0.02 ± 0.65	0.00 [0.00, 0.00]	0.00 – 23.00	0.0
**68**	Cancer : diagnostic duration (second time)	Continuous	0.08 ± 1.26	0.00 [0.00, -0.00]	-0.00 – 39.50	13.0
**69**	Fresh fruit intake	Continuous	2.26 ± 1.37	2.00 [1.00, 3.00]	0.00 – 10.00	100373.0
**70**	Wearing glasses or lenses : duration from when started	Continuous	28.21 ± 19.15	26.00 [12.00, 45.00]	-0.00 – 77.00	100291.0
**71**	Stroke : diagnostic age	Continuous	0.60 ± 6.08	0.00 [0.00, 0.00]	0.00 – 75.00	100291.0
**72**	Smoking : age when started	Continuous	3.53 ± 7.06	0.00 [0.00, 0.00]	0.00 – 40.00	100291.0
**73**	Arm fat-free mass (right)	Continuous	2.89 ± 0.81	2.60 [2.20, 3.60]	1.40 – 5.60	0.0
**74**	Depression : duration from first episode	Continuous	12.03 ± 15.65	0.00 [0.00, 23.00]	-0.00 – 64.00	513.0
**75**	Non-cancer illness: diagnostic age (fourth time)	Continuous	9.07 ± 19.42	0.00 [0.00, 0.00]	0.00 – 77.60	13.0
**76**	Arm predicted mass (left)	Continuous	2.72 ± 0.79	2.50 [2.00, 3.40]	1.40 – 5.30	0.0
**77**	Leg fat-free mass (right)	Continuous	8.99 ± 1.96	8.60 [7.30, 10.50]	5.40 – 16.60	0.0
**78**	Unusual or psychotic experience : diagnostic duration	Continuous	3.67 ± 52.43	0.00 [0.00, -0.00]	-0.00 – 1068.00	530.0
**79**	Body fat percentage	Continuous	29.93 ± 7.98	29.50 [24.10, 35.80]	8.20 – 56.00	0.0
**80**	Chronic bronchitis : diagnostic duration	Continuous	0.11 ± 1.95	0.00 [0.00, -0.00]	-0.00 – 48.00	0.0
**81**	Dried fruit intake	Continuous	0.90 ± 1.44	0.50 [0.00, 1.00]	0.00 – 15.00	100373.0
**82**	Lung cancer (not mesothelioma) : diagnostic age	Continuous	0.01 ± 0.74	0.00 [0.00, 0.00]	0.00 – 45.00	0.0
**83**	Diastolic blood pressure, automated reading	Continuous	81.00 ± 10.26	81.00 [74.00, 88.00]	41.00 – 121.00	0.0
**84**	Whole body fat-free mass	Continuous	53.34 ± 11.32	50.40 [43.60, 62.88]	33.10 – 90.40	0.0
**85**	Sarcoidosis : diagnostic age	Continuous	0.09 ± 2.01	0.00 [0.00, 0.00]	0.00 – 60.00	0.0
**86**	Duration of moderate activity	Continuous	61.66 ± 63.35	45.00 [20.00, 60.00]	0.00 – 600.00	100291.0
**87**	Angina : diagnostic age	Continuous	1.02 ± 7.63	0.00 [0.00, 0.00]	0.00 – 78.00	100291.0
**88**	Depression : age at first episode	Continuous	18.81 ± 21.09	10.00 [0.00, 37.75]	0.00 – 75.00	513.0
**89**	Hayfever or allergic rhinitis : diagnostic duration	Continuous	6.15 ± 14.99	0.00 [0.00, -0.00]	-0.00 – 69.00	0.0
**90**	Operation : age when took place (fourth time)	Continuous	5.76 ± 15.87	0.00 [0.00, 0.00]	0.00 – 77.80	13.0
**91**	Emphysema/Chronic bronchitis : diagnostic duration	Continuous	0.13 ± 2.30	0.00 [0.00, -0.00]	-0.00 – 65.00	100291.0
**92**	Bread intake	Continuous	10.07 ± 7.26	9.00 [5.00, 14.00]	0.00 – 60.00	100373.0
**93**	Trunk fat percentage	Continuous	29.68 ± 7.46	29.60 [24.80, 34.60]	3.00 – 59.50	0.0
**94**	Depression : duration from last episode	Continuous	6.72 ± 10.87	0.00 [0.00, 10.00]	-0.00 – 57.00	513.0
**95**	Operation : duration of taking place (first time)	Continuous	20.99 ± 19.87	15.60 [2.10, 35.50]	0.00 – 73.50	13.0
**96**	Cataract : diagnostic duration	Continuous	0.51 ± 2.63	0.00 [0.00, -0.00]	-0.00 – 63.00	100291.0
**97**	Birth weight	Continuous	3.37 ± 0.59	3.37 [3.06, 3.69]	0.91 – 5.90	0.0
**98**	Depression : age at last episode	Continuous	24.13 ± 25.37	18.00 [0.00, 50.00]	0.00 – 77.00	513.0

**99**	Non-cancer illness: diagnostic duration (sixth time)	Continuous	3.26 ± 11.67	0.00 [0.00, -0.00]	-0.00 – 71.70	13.0
**100**	Smoking : duration	Continuous	5.00 ± 11.20	0.00 [0.00, 0.00]	0.00 – 62.00	100291.0
**101**	Asthma : diagnostic age by doctor	Continuous	2.73 ± 10.22	0.00 [0.00, 0.00]	0.00 – 72.00	0.0
**102**	Impedance of arm (left)	Continuous	336.93 ± 55.87	335.00 [292.00, 378.00]	197.00 – 548.00	0.0
**103**	Non-cancer illness: diagnostic duration (fourth time)	Continuous	6.07 ± 14.93	0.00 [0.00, -0.00]	-0.00 – 73.50	13.0
**104**	Impedance of arm (right)	Continuous	329.51 ± 55.47	327.00 [284.00, 370.00]	192.00 – 948.00	0.0
**105**	Forced expiratory volume in 1-second (FEV1)	Continuous	3.06 ± 0.82	2.98 [2.53, 3.52]	0.20 – 21.92	0.0
**106**	Operation : duration of taking place (sixth time)	Continuous	-5.62 ± 13.14	-5.38 [-13.70, 0.00]	-36.15 – 87.68	13.0
**107**	Sarcoidosis : diagnostic duration	Continuous	0.05 ± 1.30	0.00 [0.00, -0.00]	-0.00 – 46.00	0.0
**108**	Trunk predicted mass	Continuous	28.48 ± 5.75	26.90 [23.60, 33.30]	14.80 – 46.90	0.0
**109**	Unusual or psychotic experience : diagnostic age	Continuous	-1.17 ± 49.75	0.00 [0.00, 0.00]	-999.00 – 67.00	530.0
**110**	Wearing glasses or lenses : age when started	Continuous	29.91 ± 18.45	30.00 [13.00, 46.00]	0.00 – 77.00	100291.0
**111**	Non-cancer illness: diagnostic duration (fifth time)	Continuous	4.31 ± 13.25	0.00 [0.00, -0.00]	-0.00 – 71.50	13.0
**112**	Emphysema : diagnostic duration	Continuous	0.00 ± 0.11	0.00 [0.00, -0.00]	-0.00 – 5.00	0.0
**113**	Operation : duration of taking place (second time)	Continuous	13.51 ± 18.55	1.90 [0.00, 24.18]	0.00 – 73.70	13.0
**114**	Trunk fat mass	Continuous	12.78 ± 4.62	12.30 [9.50, 15.40]	1.30 – 37.90	0.0
**115**	Heel bone mineral density (BMD) T-score, automated (left)	Continuous	-0.27 ± 1.19	-0.36 [-1.04, 0.39]	-5.53 – 8.27	0.0
**116**	Forced vital capacity (FVC)	Continuous	3.99 ± 1.04	3.87 [3.28, 4.60]	0.53 – 28.03	0.0
**117**	Hay fever, rhinitis or eczema : diagnostic age	Continuous	5.53 ± 12.65	0.00 [0.00, 0.00]	0.00 – 72.00	100291.0
**118**	Arm predicted mass (right)	Continuous	2.70 ± 0.77	2.40 [2.00, 3.30]	1.30 – 5.30	0.0
**119**	Leg fat mass (left)	Continuous	3.93 ± 1.62	3.70 [2.80, 4.70]	0.80 – 26.30	0.0
**120**	Asthma : diagnostic age	Continuous	3.11 ± 10.78	0.00 [0.00, 0.00]	0.00 – 69.00	100291.0
**121**	Non-cancer illness: diagnostic age (seventh time)	Continuous	2.74 ± 11.06	0.00 [0.00, 0.00]	0.00 – 78.50	13.0
**122**	Leg fat percentage (left)	Continuous	30.51 ± 10.05	31.90 [21.10, 39.00]	9.00 – 61.70	0.0
**123**	Operation : duration of taking place (fifth time)	Continuous	1.37 ± 6.95	0.00 [0.00, -0.00]	-0.00 – 69.50	13.0
**124**	Emphysema : diagnostic age	Continuous	0.05 ± 1.83	0.00 [0.00, 0.00]	0.00 – 68.00	0.0
**125**	Operation : age when took place (third time)	Continuous	11.36 ± 20.36	0.00 [0.00, 14.95]	0.00 – 78.10	13.0
**126**	Operation : age when took place (second time)	Continuous	20.65 ± 23.44	7.50 [0.00, 42.05]	0.00 – 78.20	13.0
**127**	Peak expiratory flow (PEF)	Continuous	430.13 ± 134.96	417.50 [345.00, 513.75]	8.00 – 2850.00	0.0
**128**	Chronic Obstructive Pulmonary Disease : diagnostic age	Continuous	0.17 ± 3.16	0.00 [0.00, 0.00]	0.00 – 69.00	0.0
**129**	Cancer : diagnostic age (first time)	Continuous	6.78 ± 18.58	0.00 [0.00, 0.00]	0.00 – 76.40	13.0
**130**	Salad / raw vegetable intake	Continuous	2.23 ± 1.95	2.00 [1.00, 3.00]	0.00 – 25.00	100373.0
**131**	Systolic blood pressure, automated reading	Continuous	135.61 ± 17.95	134.00 [123.00, 147.00]	92.00 – 233.00	0.0
**132**	Operation : age when took place (first time)	Continuous	30.42 ± 22.51	31.55 [7.50, 49.90]	0.00 – 76.90	13.0

**133**	Glaucoma : diagnostic duration	Continuous	0.19 ± 1.92	0.00 [0.00, -0.00]	-0.00 – 63.00	100291.0
**134**	Chronic bronchitis : diagnostic age	Continuous	0.19 ± 2.98	0.00 [0.00, 0.00]	0.00 – 62.00	0.0
**135**	Arm fat percentage (right)	Continuous	27.84 ± 9.22	26.40 [20.40, 34.30]	6.40 – 72.00	0.0
**136**	Impedance of leg (left)	Continuous	250.11 ± 33.02	248.00 [227.00, 272.00]	106.00 – 363.00	0.0
**137**	Heart attack : diagnostic duration	Continuous	0.16 ± 1.63	0.00 [0.00, -0.00]	-0.00 – 43.00	100291.0
**138**	Cancer : diagnostic duration (first time)	Continuous	1.14 ± 4.30	0.00 [0.00, -0.00]	-0.00 – 37.50	13.0
**139**	Operation : age when took place (sixth time)	Continuous	-6.93 ± 18.07	-9.55 [-19.12, 0.00]	-37.30 – 94.40	13.0
**140**	Non-cancer illness: diagnostic age (eighth time)	Continuous	1.98 ± 9.54	0.00 [0.00, 0.00]	0.00 – 77.30	13.0
**141**	Operation : age when took place (fifth time)	Continuous	2.69 ± 11.29	0.00 [0.00, 0.00]	0.00 – 77.30	13.0
**142**	Pulse wave Arterial Stiffness index	Continuous	9.52 ± 2.86	9.37 [7.44, 11.26]	1.35 – 25.00	0.0
**143**	Non-cancer illness: diagnostic duration (eighth time)	Continuous	1.83 ± 9.10	0.00 [0.00, -0.00]	-0.00 – 72.80	13.0
**144**	Heart attack : diagnostic age	Continuous	0.91 ± 7.28	0.00 [0.00, 0.00]	0.00 – 71.00	100291.0
**145**	Non-cancer illness: diagnostic age (fifth time)	Continuous	5.71 ± 16.03	0.00 [0.00, 0.00]	0.00 – 76.50	13.0
**146**	Whole body water mass	Continuous	39.01 ± 8.27	36.80 [31.90, 45.90]	24.20 – 66.20	0.0
**147**	Non-cancer illness: diagnostic age (third time)	Continuous	14.57 ± 23.47	0.00 [0.00, 27.65]	0.00 – 79.50	13.0
**148**	diabetes-related eye disease : diagnostic age	Continuous	0.26 ± 3.83	0.00 [0.00, 0.00]	0.00 – 73.00	100291.0
**149**	Loss of vision due to injury or trauma: diagnostic age	Continuous	0.17 ± 2.98	0.00 [0.00, 0.00]	0.00 – 68.00	100291.0
**150**	Tea intake	Continuous	3.44 ± 2.68	3.00 [1.00, 5.00]	0.00 – 28.00	100373.0
**151**	Non-cancer illness: diagnostic duration (first time)	Continuous	13.05 ± 16.56	6.00 [0.00, 19.50]	-0.00 – 76.60	13.0
**152**	Cancers count : self-reported number	Continuous	0.06 ± 0.25	0.00 [0.00, 0.00]	0.00 – 2.00	0.0

Supplemantary Table 4. Descriptive table for categorical variables in the Main subset.

	Feature	Type	Categories / Levels	mode	coding ID
0	Smoking status	Categorical	0.0: 64.6%; 1.0: 30.1%; 2.0: 5.3%	0.0	90.0
1	Comparative height size at age 10	Categorical	3.0: 51.5%; 5.0: 28.6%; 1.0: 19.9%	3.0	100429.0
2	Feelings of anxiety/nervousness lately	Categorical	1.0: 77.1%; 2.0: 20.3%; 3.0: 1.3%; 4.0: 1.3%	1.0	504.0
3	Years of education	Categorical	19.0: 73.4%; 15.0: 10.1%; 10.0: 9.4%; 13.0: 4.3%; 7.0: 2.7%	19.0	100305.0
4	Diabetes : diagnosis	Categorical	0.0: 98.1%; 1.0: 1.9%	0.0	100349.0
5	Feelings of foreboding lately	Categorical	1.0: 87.5%; 2.0: 10.7%; 3.0: 0.9%; 4.0: 0.8%	1.0	504.0
6	Cancer : diagnosis	Categorical	0.0: 94.5%; 1.0: 5.5%	0.0	100603.0
7	Poor appetite and overeating lately	Categorical	1.0: 85.5%; 2.0: 11.2%; 3.0: 1.8%; 4.0: 1.5%	1.0	504.0
8	Poultry intake	Categorical	3.0: 47.8%; 2.0: 34.4%; 1.0: 9.4%; 0.0: 6.8%; 4.0: 1.5%; 5.0: 0.2%	3.0	100377.0
9	Financial situation satisfaction	Categorical	2.0: 48.0%; 3.0: 29.2%; 1.0: 18.6%; 4.0: 3.0%; 5.0: 0.8%; 6.0: 0.4%	2.0	100478.0
10	Belief in a personal conspiracy	Categorical	0.0: 99.5%; 1.0: 0.5%	0.0	502.0
11	Felt loved as a child	Categorical	4.0: 57.3%; 3.0: 24.9%; 2.0: 13.7%; 1.0: 3.2%; 0.0: 0.8%	4.0	532.0
12	Contemplated self-harm	Categorical	0.0: 86.2%; 1.0: 7.2%; 2.0: 6.6%	0.0	535.0
13	Injury Caused by alcohol consumption	Categorical	0.0: 96.4%; 1.0: 3.1%; 2.0: 0.5%	0.0	524.0
14	Combat/War-Zone Involvement	Categorical	0.0: 97.0%; 1.0: 3.0%; 2.0: 0.0%	0.0	533.0
15	Loud music exposure frequency	Categorical	14.0: 88.6%; 12.0: 4.9%; 11.0: 4.1%; 13.0: 2.4%	14.0	100637.0
16	Oily fish intake	Categorical	2.0: 41.1%; 1.0: 34.4%; 3.0: 16.3%; 0.0: 7.6%; 4.0: 0.5%; 5.0: 0.1%	2.0	100377.0
17	Worried more than others in similar situations	Categorical	0.0: 77.0%; 1.0: 23.0%	0.0	502.0
18	Noisy workplace	Categorical	14.0: 83.0%; 11.0: 7.6%; 13.0: 5.1%; 12.0: 4.4%	14.0	100637.0
19	Other serious medical conditions and disabilities	Categorical	0.0: 84.8%; 1.0: 15.2%	0.0	100603.0
20	Falls in the last year	Categorical	1.0: 86.2%; 2.0: 10.4%; 3.0: 3.5%	1.0	100539.0
21	Physically abused by family as a child	Categorical	0.0: 80.2%; 1.0: 12.1%; 2.0: 6.5%; 3.0: 0.8%; 4.0: 0.4%	0.0	532.0
22	Victim of sexual assault	Categorical	0.0: 86.0%; 1.0: 13.9%; 2.0: 0.1%	0.0	533.0
23	Non-consensual sexual Interference by ex-/partners	Categorical	0.0: 95.3%; 1.0: 2.6%; 2.0: 1.5%; 4.0: 0.3%; 3.0: 0.3%	0.0	532.0
24	Feelings of restlessness lately	Categorical	1.0: 89.9%; 2.0: 8.3%; 3.0: 1.1%; 4.0: 0.7%	1.0	504.0
25	Sexually molested as a child	Categorical	0.0: 92.1%; 1.0: 4.0%; 2.0: 3.0%; 3.0: 0.4%; 4.0: 0.4%	0.0	532.0
26	Activities Avoided Due to Recent Stress	Categorical	0.0: 83.3%; 1.0: 13.3%; 2.0: 2.0%; 3.0: 1.2%; 4.0: 0.3%	0.0	534.0
27	Qualifications	Categorical	1.0: 51.0%; 3.0: 18.5%; 2.0: 13.8%; 5.0: 5.0%; 6.0: 4.7%; -7.0: 3.8%; 4.0: 3.3%	1.0	100305.0
28	Encoded anonymised participant ID	Categorical	5.0: 73.4%; 6.0: 10.1%; 2.0: 9.4%; 3.0: 4.3%; 1.0: 2.7%	5.0	100305.0
29	Confiding Relationships in Adulthood	Categorical	4.0: 57.9%; 3.0: 17.2%; 2.0: 15.4%; 0.0: 6.5%; 1.0: 3.0%	4.0	532.0
30	Spread type	Categorical	1.0: 55.3%; 3.0: 34.4%; 0.0: 10.3%	1.0	100388.0

31	Hot drink temperature	Categorical	2.0: 66.9%; 1.0: 17.1%; 3.0: 15.2%; 4.0: 0.9%	2.0	100398.0
32	Mineral and other dietary supplements	Categorical	-7.0: 64.0%; 1.0: 25.7%; 2.0: 5.2%; 3.0: 2.4%; 5.0: 1.3%; 4.0: 1.0%; 6.0: 0.4%	-7.0	100630.0
33	Blood Clot, DVT, and Respiratory Conditions	Categorical	-7.0: 66.5%; 9.0: 20.6%; 8.0: 11.3%; 5.0: 1.1%; 6.0: 0.4%; 7.0: 0.2%	-7.0	100610.0
34	Exclude eggs, dairy, wheat, sugar from diet	Categorical	5.0: 77.5%; 4.0: 18.2%; 1.0: 1.8%; 3.0: 1.8%; 2.0: 0.6%	5.0	100385.0
35	Medication for pain relief, constipation, heartburn	Categorical	-7.0: 63.7%; 2.0: 11.3%; 1.0: 9.1%; 3.0: 8.4%; 5.0: 5.4%; 6.0: 1.1%; 4.0: 1.0%	-7.0	100628.0
36	Facial ageing	Categorical	1.0: 78.3%; 3.0: 20.8%; 5.0: 0.9%	1.0	100435.0
37	Long-standing illness, disability or infirmity	Categorical	0.0: 77.8%; 1.0: 22.2%	0.0	100349.0
38	Changes in speed/amount of moving or speaking lately	Categorical	1.0: 96.7%; 2.0: 3.0%; 3.0: 0.2%; 4.0: 0.1%	1.0	504.0
39	Long-term loss of interest in activities	Categorical	0.0: 65.4%; 1.0: 34.6%	0.0	503.0
40	Mental distress affecting daily activities	Categorical	0.0: 69.4%; 1.0: 30.6%	0.0	502.0
41	Sought help for mental distress	Categorical	0.0: 64.2%; 1.0: 35.8%	0.0	502.0
42	Cannabis usage	Categorical	0.0: 76.6%; 1.0: 10.5%; 2.0: 5.7%; 3.0: 4.6%; 4.0: 2.7%	0.0	526.0
43	Felt upset by reminders of past stress	Categorical	0.0: 66.3%; 1.0: 26.8%; 2.0: 4.3%; 3.0: 2.2%; 4.0: 0.5%	0.0	534.0
44	Non-oily fish intake	Categorical	2.0: 52.1%; 1.0: 28.3%; 3.0: 15.2%; 0.0: 3.9%; 4.0: 0.5%; 5.0: 0.1%	2.0	100377.0
45	Race	Categorical	1.0: 98.1%; 3.0: 1.0%; 6.0: 0.3%; 2.0: 0.3%; 4.0: 0.3%	1.0	1001.0
46	Excessive worrying lately	Categorical	1.0: 74.3%; 2.0: 22.6%; 3.0: 1.7%; 4.0: 1.4%	1.0	504.0
47	Physical violence from ex-/partner	Categorical	0.0: 87.7%; 1.0: 6.3%; 2.0: 4.4%; 4.0: 0.9%; 3.0: 0.6%	0.0	532.0
48	Comparative body size at age 10	Categorical	3.0: 51.8%; 1.0: 33.4%; 5.0: 14.8%	3.0	100428.0
49	Processed meat intake	Categorical	1.0: 31.7%; 2.0: 30.2%; 3.0: 25.3%; 0.0: 9.9%; 4.0: 2.3%; 5.0: 0.6%	1.0	100377.0
50	Period of extreme irritability	Categorical	0.0: 77.1%; 1.0: 22.9%	0.0	502.0
51	Diminished pleasure in activities lately	Categorical	1.0: 87.5%; 2.0: 10.5%; 3.0: 1.3%; 4.0: 0.7%	1.0	504.0
52	Feelings of tiredness/low energy lately	Categorical	1.0: 58.4%; 2.0: 34.3%; 3.0: 4.6%; 4.0: 2.7%	1.0	504.0
53	Lamb/mutton intake	Categorical	1.0: 60.2%; 2.0: 21.8%; 0.0: 16.0%; 3.0: 2.0%	1.0	100377.0
54	Major dietary changes in the last 5 years	Categorical	0.0: 66.6%; 2.0: 27.9%; 1.0: 5.5%	0.0	100400.0
55	Having thoughts of self-harm lately	Categorical	1.0: 97.3%; 2.0: 2.2%; 3.0: 0.3%; 4.0: 0.2%	1.0	504.0
56	Wheeze or whistling in the chest in last year	Categorical	0.0: 85.1%; 1.0: 14.9%	0.0	100349.0
57	Belief in unreal communications	Categorical	0.0: 99.4%; 1.0: 0.6%	0.0	502.0
58	Vascular/heart problems	Categorical	-7.0: 82.9%; 4.0: 14.9%; 3.0: 0.8%; 1.0: 0.8%; 2.0: 0.7%	-7.0	100605.0
59	Pork intake	Categorical	1.0: 59.7%; 2.0: 22.1%; 0.0: 15.7%; 3.0: 2.5%; 4.0: 0.1%	1.0	100377.0
60	Weight change in last year	Categorical	0.0: 62.6%; 2.0: 22.9%; 3.0: 14.6%	0.0	100540.0
61	Pain type(s) experienced in last month	Categorical	-7.0: 46.7%; 1.0: 20.0%; 3.0: 12.1%; 4.0: 10.4%; 7.0: 5.7%; 5.0: 2.1%; 6.0: 2.0%; 8.0: 0.6%; 2.0: 0.4%	-7.0	100553.0
62	Difficulty relaxing lately	Categorical	1.0: 75.7%; 2.0: 20.5%; 3.0: 2.0%; 4.0: 1.8%	1.0	504.0
63	Salt added to food	Categorical	1.0: 63.8%; 2.0: 24.0%; 3.0: 10.1%; 4.0: 2.2%	1.0	100394.0
64	Alcohol intake	Categorical	3.0: 32.1%; 4.0: 31.1%; 2.0: 19.0%; 1.0: 11.8%; 0.0: 6.0%	3.0	521.0

65	Variation in diet	Categorical	2.0: 57.1%; 1.0: 35.1%; 3.0: 7.8%	2.0	100401.0
66	Known person advocated alcohol reduction	Categorical	0.0: 92.3%; 2.0: 3.9%; 1.0: 3.8%	0.0	524.0
67	Able to pay rent/mortgage as an adult	Categorical	4.0: 88.0%; 3.0: 6.7%; 0.0: 2.8%; 2.0: 1.9%; 1.0: 0.5%	4.0	532.0
68	heard an un-real voice	Categorical	0.0: 98.7%; 1.0: 1.3%	0.0	502.0
69	Beef intake	Categorical	1.0: 45.1%; 2.0: 32.8%; 0.0: 11.9%; 3.0: 10.0%; 4.0: 0.2%	1.0	100377.0
70	Family relationship satisfaction	Categorical	2.0: 50.6%; 3.0: 23.0%; 1.0: 21.4%; 4.0: 3.9%; 5.0: 0.6%; 6.0: 0.4%	2.0	100478.0
71	seen an un-real vision	Categorical	0.0: 97.6%; 1.0: 2.4%	0.0	502.0
72	Trouble focusing on tasks lately	Categorical	1.0: 86.2%; 2.0: 11.8%; 3.0: 1.2%; 4.0: 0.7%	1.0	504.0
73	Sleep Disruptions: Difficulty Falling or Staying Asleep	Categorical	1.0: 56.0%; 2.0: 32.6%; 3.0: 5.8%; 4.0: 5.6%	1.0	504.0
74	Overall health rating	Categorical	2.0: 60.7%; 1.0: 29.1%; 3.0: 9.4%; 4.0: 0.8%	2.0	100508.0
75	Addiction to any substance or behaviour	Categorical	0.0: 94.9%; 1.0: 5.1%	0.0	502.0
76	Work/job satisfaction	Categorical	7.0: 36.6%; 2.0: 28.1%; 3.0: 23.4%; 1.0: 7.1%; 4.0: 3.8%; 5.0: 0.7%; 6.0: 0.4%	7.0	100479.0
77	Thought life was not worth living	Categorical	0.0: 73.4%; 2.0: 13.7%; 1.0: 12.9%	0.0	535.0
78	Serious Life-Threatening Accident	Categorical	0.0: 90.9%; 1.0: 8.8%; 2.0: 0.3%	0.0	533.0
79	Other prescription medications	Categorical	0.0: 64.8%; 1.0: 35.2%	0.0	100603.0
80	General happiness	Categorical	2.0: 48.9%; 3.0: 34.6%; 1.0: 13.2%; 4.0: 2.7%; 5.0: 0.4%; 6.0: 0.2%	2.0	537.0
81	Home area population density - urban or rural	Categorical	5.0: 82.3%; 6.0: 8.0%; 7.0: 5.3%; 8.0: 3.3%; 11.0: 0.8%; 12.0: 0.2%; 16.0: 0.1%; 13.0: 0.0%	5.0	91.0
82	Feelings of inadequacy lately	Categorical	1.0: 85.2%; 2.0: 12.4%; 3.0: 1.2%; 4.0: 1.2%	1.0	504.0
83	Belief that own life is meaningful	Categorical	4.0: 57.9%; 3.0: 21.3%; 5.0: 16.6%; 2.0: 3.2%; 1.0: 1.1%	4.0	538.0
84	Milk type used	Categorical	2.0: 66.3%; 3.0: 22.5%; 1.0: 4.4%; 4.0: 3.3%; 0.0: 2.5%; 5.0: 0.9%	2.0	100387.0
85	Witnessed sudden violent death	Categorical	0.0: 85.5%; 1.0: 13.9%; 2.0: 0.6%	0.0	533.0
86	Easily annoyed or irritable lately	Categorical	1.0: 77.0%; 2.0: 20.8%; 3.0: 1.5%; 4.0: 0.7%	1.0	504.0
87	General happiness with own health	Categorical	2.0: 44.2%; 3.0: 36.9%; 1.0: 11.7%; 4.0: 5.7%; 5.0: 1.1%; 6.0: 0.4%	2.0	537.0
88	Bread type	Categorical	3.0: 67.9%; 1.0: 19.1%; 2.0: 9.7%; 4.0: 3.3%	3.0	100391.0
89	Belittlement by partner or ex-partner as an adult	Categorical	0.0: 77.3%; 1.0: 9.7%; 2.0: 8.9%; 3.0: 2.2%; 4.0: 2.0%	0.0	532.0
90	Alcohol intake frequency	Categorical	2.0: 29.3%; 3.0: 26.8%; 1.0: 21.6%; 4.0: 10.7%; 5.0: 7.4%; 6.0: 4.2%	2.0	100402.0
91	Felt hated by family member as a child	Categorical	0.0: 85.6%; 1.0: 6.3%; 2.0: 6.1%; 3.0: 1.0%; 4.0: 1.0%	0.0	532.0
92	Disturbing thoughts of past stress lately	Categorical	0.0: 75.9%; 1.0: 19.2%; 2.0: 2.6%; 3.0: 2.1%; 4.0: 0.2%	0.0	534.0
93	Feelings of depression lately	Categorical	1.0: 85.2%; 2.0: 13.2%; 3.0: 1.0%; 4.0: 0.7%	1.0	504.0
94	Difficulty stopping worry lately	Categorical	1.0: 83.1%; 2.0: 14.4%; 3.0: 1.3%; 4.0: 1.2%	1.0	504.0
95	Chest pain or discomfort	Categorical	0.0: 89.4%; 1.0: 10.6%	0.0	100349.0
96	Friendships satisfaction	Categorical	2.0: 56.7%; 3.0: 26.2%; 1.0: 15.4%; 4.0: 1.5%; 5.0: 0.1%; 6.0: 0.1%	2.0	100478.0
97	Long-lasting sadness or depression	Categorical	0.0: 50.7%; 1.0: 49.3%	0.0	503.0
98	Period of mania / excitability	Categorical	0.0: 96.3%; 1.0: 3.7%	0.0	502.0

99	Maternal smoking around birth	Categorical	0.0: 70.9%; 1.0: 29.1%	0.0	100349.0
100	Vitamin and mineral supplements	Categorical	-7.0: 64.2%; 7.0: 15.1%; 4.0: 10.3%; 3.0: 4.2%; 2.0: 3.5%; 1.0: 1.6%; 6.0: 0.8%; 5.0: 0.4%	-7.0	100629.0
101	Cheese intake	Categorical	3.0: 49.3%; 2.0: 19.8%; 1.0: 13.8%; 4.0: 11.8%; 5.0: 4.0%; 0.0: 1.4%	3.0	100377.0
102	Long-term worry and tension	Categorical	0.0: 75.0%; 1.0: 25.0%	0.0	502.0
103	Victim of physically violent crime	Categorical	0.0: 81.8%; 1.0: 17.7%; 2.0: 0.5%	0.0	533.0
104	Life-threatening illness	Categorical	0.0: 88.2%; 1.0: 10.3%; 2.0: 1.5%	0.0	533.0
105	Ever self-harmed	Categorical	0.0: 96.3%; 1.0: 3.7%	0.0	503.0
106	Handedness (chirality/laterality)	Categorical	1.0: 89.2%; 2.0: 9.2%; 3.0: 1.6%	1.0	100430.0
107	Childhood Health: The Role of a Supportive Caregiver	Categorical	4.0: 87.5%; 3.0: 8.4%; 0.0: 1.9%; 2.0: 1.8%; 1.0: 0.5%	4.0	532.0

Supplementary Table 5. Grey matter health prediction performances: Grey matter health prediction performance across different subsets/algorithms including main subset (n = 3706), replication subset (n = 4202), and variable-restricted subset (n = 7736). P-values were estimated using permutation testing with 500 permutations and corrected for multiple comparisons using False Discovery Rate (FDR). The smallest p-value obtainable with this procedure is approximately 0.002. The implementation of permutation testing corresponds to a one-tailed (one-sided) test.

**Algorithm**	Random Forest			SVR(Linear)	Ridge regression
**Subset**	**Main**	Replication	Variables-restricted	Main	Main
**Mean Absolute Error (MAE)**	**4.48**	4.52	4.53	4.54	4.53
**Uncorrected p-value**	**0.034**	0.024	0.002	0.002	0.002
**Corrected p-value**	**0.035**	0.026	0.003	0.003	0.003
**Mean Squared Error (MSE)**	**32.22**	32.63	32.66	33.14	33.07
**Uncorrected p-value**	**0.006**	0.022	0.002	0.002	0.002
**Corrected p-value**	**0.007**	0.024	0.003	0.003	0.003
**root Mean Squared Error (rMSE)**	**5.67**	5.71	5.71	5.75	5.75
**Uncorrected p-value**	**0.004**	0.044	0.002	0.002	0.002
**Corrected p-value**	**0.005**	0.044	0.003	0.003	0.003
**r^2**	**0.05**	0.05	0.06	0.02	0.03
**Uncorrected p-value**	**0.002**	0.004	0.002	0.002	0.002
**Corrected p-value**	**0.003**	0.005	0.003	0.003	0.003
**Pearson Correlation (r)**	**0.23**	0.23	0.24	0.16	0.17
**Uncorrected p-value**	**0.002**	0.004	0.002	0.002	0.002
**Corrected p-value**	**0.003**	0.005	0.003	0.003	0.003

Supplementary Table 6. Feature importance ranking of all exposome variables in the main subset (*n* = 3706) based on different algorithms.

Category	Title	Ranking RF	Ranking SVR	Ranking Ridge	RF (mean absolute SHAP value)	SVR (mean absolute SHAP value)	Ridge (mean absolute SHAP value)
Cardiovascular health	High blood pressure : diagnostic age	1	9	4	0.222	0.065	0.060
Eye/Vision impairment	Loss of vision due to injury or trauma: diagnostic age	2	181	182	0.194	0.006	0.003
Body composition and body morphology	Sitting height	3	46	42	0.177	0.030	0.024
Diabetes	Diabetes disease : diagnostic duration	4	62	47	0.161	0.024	0.023
Cardiovascular health	mean absolute SHAP value	5	6	8	0.140	0.067	0.052
Cardiovascular health	Diastolic blood pressure, automated reading	6	8	1	0.110	0.065	0.070
Cardiovascular health	Smoking : age when started	7	15	12	0.105	0.050	0.044
Diet and alcohol	Coffee intake	8	4	2	0.103	0.072	0.066
Body injuries and illnesses	Cancer : diagnostic duration (first time)	9	162	171	0.101	0.008	0.004
Cardiovascular health	Smoking : age when stopped	10	7	7	0.090	0.065	0.054
Diet and alcohol	Dried fruit intake	11	12	17	0.080	0.055	0.039
Eye/Vision impairment	Glaucoma : diagnostic age	12	76	111	0.079	0.022	0.011
Diet and alcohol	Cereal intake	13	14	5	0.069	0.052	0.055
Diabetes	Diabetes-related eye disease : diagnostic duration	14	220	203	0.063	0.002	0.002
Bone health	Heel bone mineral density (BMD) T-score, automated (right)	15	57	14	0.061	0.026	0.042
Bone health	Heel bone mineral density (BMD) T-score, automated (left)	16	88	51	0.056	0.019	0.022
Diabetes	Diabetes disease : diagnostic age	17	20	20	0.053	0.044	0.033
Mental health and socio-affective factors	Unusual or psychotic experience : diagnostic age	18	226	214	0.052	0.002	0.001
Body injuries and illnesses	Operation : age when took place (first time)	19	67	9	0.049	0.023	0.052
Diet and alcohol	Alcohol intake	20	1	3	0.046	0.087	0.066
Cardiovascular health	High blood pressure : diagnostic duration	21	11	16	0.043	0.055	0.040
Body injuries and illnesses	Operation : age when took place (second time)	22	3	11	0.043	0.077	0.047
Cardiovascular health	Pulse rate	23	121	46	0.040	0.013	0.023
Diet and alcohol	Cooked vegetable intake	24	79	56	0.040	0.021	0.021
Body composition and body morphology	Hip circumference	25	98	28	0.035	0.017	0.029
Body injuries and illnesses	Cancer : diagnostic age (first time)	26	208	156	0.032	0.003	0.004
Body injuries and illnesses	Non-cancer illness: diagnostic age (first time)	27	97	18	0.032	0.017	0.037
Body injuries and illnesses	Non-cancer illness: diagnostic duration (first time)	28	44	117	0.030	0.030	0.010
Cardiovascular health	Smoking : age when started again (current smokers)	29	69	100	0.029	0.022	0.012
Body composition and body morphology	Hand grip strength (left)	30	146	106	0.029	0.009	0.011
Diet and alcohol	Bread intake	31	150	204	0.029	0.008	0.002
Body injuries and illnesses	Non-cancer illnesses : self-reported number	32	27	29	0.029	0.038	0.028

Body injuries and illnesses	Cancers count : self-reported number	33	212	219	0.029	0.002	0.001
Body composition and body morphology	Forced vital capacity (FVC)	34	35	70	0.028	0.033	0.018
Diet and alcohol	Salad / raw vegetable intake	35	96	116	0.028	0.017	0.010
Body injuries and illnesses	Operations count : self-reported number	36	63	146	0.027	0.024	0.006
Diet and alcohol	Fresh fruit intake	37	155	71	0.027	0.008	0.018
Mental health and socio-affective factors	Depression : age at last episode	38	49	169	0.026	0.028	0.004
Mental health and socio-affective factors	Unusual or psychotic experience : diagnostic duration	39	230	210	0.026	0.001	0.001
Lifestyle and environment	Duration of moderate activity	40	107	72	0.025	0.015	0.018
Body injuries and illnesses	Chronic bronchitis : diagnostic age	41	133	123	0.025	0.011	0.009
Mental health and socio-affective factors	Cannabis : age when last took	42	108	40	0.024	0.015	0.025
Eye/Vision impairment	Macular degeneration : diagnostic age	43	159	246	0.024	0.008	0.0004
Cardiovascular health	Angina : diagnostic duration	44	207	251	0.024	0.003	0.0003
Body injuries and illnesses	Operation : duration of taking place (first time)	45	61	128	0.023	0.024	0.009
Diet and alcohol	Water intake	46	21	65	0.022	0.042	0.019
Early life factors	Birth weight	47	193	60	0.022	0.004	0.020
Diet and alcohol	Alcohol intake frequency	48	2	6	0.022	0.079	0.054
Body composition and body morphology	Hand grip strength (right)	49	130	243	0.021	0.012	0.0006
Cardiovascular health	Systolic blood pressure, automated reading	50	119	39	0.021	0.013	0.025
Cardiovascular health	Pulse wave Arterial Stiffness index	51	82	227	0.020	0.020	0.001
Body composition and body morphology	Forced expiratory volume in 1-second (FEV1)	52	50	49	0.020	0.028	0.022
Eye/Vision impairment	Wearing glasses or lenses : age when started	53	41	82	0.020	0.032	0.015
Body injuries and illnesses	Emphysema : diagnostic age	54	245	260	0.020	0.0009	0.0
Body injuries and illnesses	Non-cancer illness: diagnostic duration (second time)	55	93	95	0.020	0.018	0.013
Body injuries and illnesses	Non-cancer illness: diagnostic duration (third time)	56	16	36	0.020	0.045	0.026
Body composition and body morphology	Impedance of arm (left)	57	89	90	0.019	0.018	0.014
Body composition and body morphology	Waist circumference	58	66	126	0.018	0.023	0.009
Body composition and body morphology	Impedance of leg (right)	59	139	212	0.018	0.010	0.001
Body injuries and illnesses	Non-cancer illness: diagnostic duration (eighth time)	60	86	228	0.018	0.019	0.001
Body composition and body morphology	Peak expiratory flow (PEF)	61	40	50	0.017	0.032	0.022
Diet and alcohol	Known person advocated alcohol reduction	62	10	26	0.017	0.059	0.030
Diet and alcohol	Tea intake	63	124	104	0.017	0.013	0.011
Body injuries and illnesses	Asthma : diagnostic age by doctor	64	75	55	0.017	0.022	0.021
Eye/Vision impairment	Cataract : diagnostic age	65	148	164	0.016	0.009	0.004
Body injuries and illnesses	Non-cancer illness: diagnostic age (third time)	66	24	92	0.016	0.041	0.014
Cardiovascular health	Pulse rate, automated reading	67	91	22	0.016	0.018	0.030
Cardiovascular health	Stroke : diagnostic duration	68	188	259	0.016	0.004	0.0
Body injuries and illnesses	Operation : duration of taking place (fourth time)	69	34	67	0.016	0.033	0.018
Body injuries and illnesses	Asthma : diagnostic age	70	32	83	0.016	0.034	0.015

Body injuries and illnesses	Non-cancer illness: diagnostic age (fourth time)	71	147	230	0.016	0.009	0.001
Body injuries and illnesses	Sarcoidosis : diagnostic duration	72	211	206	0.016	0.003	0.002
Eye/Vision impairment	Wearing glasses or lenses : duration from when started	73	47	84	0.016	0.030	0.015
Body injuries and illnesses	Operation : duration of taking place (second time)	74	135	155	0.016	0.011	0.005
Mental health and socio-affective factors	Depression : duration from first episode	75	231	112	0.016	0.001	0.011
Body composition and body morphology	Impedance of arm (right)	76	111	87	0.015	0.015	0.015
Body injuries and illnesses	Emphysema : diagnostic duration	77	215	240	0.015	0.002	0.0007
Body injuries and illnesses	Chronic bronchitis : diagnostic age	78	209	232	0.015	0.003	0.0009
Body injuries and illnesses	Sarcoidosis : diagnostic age	79	243	241	0.015	0.001	0.0007
Mental health and socio-affective factors	Depression : duration from last episode	80	168	98	0.014	0.007	0.012
Cardiovascular health	Stroke : diagnostic age	81	225	234	0.014	0.002	0.0009
Eye/Vision impairment	Standard PRS for age-related macular degeneration (AMD)	82	186	127	0.014	0.005	0.009
Body injuries and illnesses	Hay fever, rhinitis or eczema : diagnostic age	83	71	62	0.014	0.022	0.020
Body injuries and illnesses	Tuberculosis : diagnostic age	84	229	208	0.014	0.002	0.001
Mental health and socio-affective factors	Depression : age at first episode	85	53	59	0.014	0.027	0.021
Body injuries and illnesses	Non-cancer illness: diagnostic duration (fourth time)	86	54	205	0.014	0.027	0.002
Body injuries and illnesses	Hay fever, rhinitis or eczema : diagnostic duration	87	117	76	0.013	0.014	0.017
Body injuries and illnesses	Non-cancer illness: diagnostic age (second time)	88	78	132	0.013	0.021	0.008
Body injuries and illnesses	Operation : age when took place (fifth time)	89	42	33	0.013	0.031	0.027
Eye/Vision impairment	Glaucoma : diagnostic duration	90	236	218	0.013	0.001	0.001
Mental health and socio-affective factors	Cannabis : duration from when last took	91	105	221	0.013	0.016	0.001
Body injuries and illnesses	Operation : age when took place (sixth time)	92	258	255	0.013	0.0001	0.0001
Body injuries and illnesses	Non-cancer illness: diagnostic duration (fifth time)	93	83	75	0.013	0.020	0.017
Body injuries and illnesses	Chronic Obstructive Pulmonary Disease : diagnostic duration	94	210	225	0.012	0.003	0.001
Body injuries and illnesses	Tuberculosis : diagnostic duration	95	237	200	0.012	0.001	0.002
Body injuries and illnesses	Asthma : diagnostic (by doctor) duration	96	172	172	0.012	0.007	0.004
Body injuries and illnesses	Hayfever or allergic rhinitis : diagnostic age	97	228	191	0.012	0.002	0.003
Body injuries and illnesses	Operation : duration of taking place (fifth time)	98	52	81	0.012	0.028	0.016
Body injuries and illnesses	Operation : duration of taking place (third time)	99	112	154	0.011	0.014	0.005
Body injuries and illnesses	Non-cancer illness: diagnostic duration (sixth time)	100	23	61	0.011	0.041	0.020
Eye/Vision impairment	Cataract : diagnostic duration	101	104	195	0.011	0.016	0.002
Body composition and body morphology	Impedance of leg (left)	102	185	153	0.011	0.005	0.005
Body injuries and illnesses	Hayfever or allergic rhinitis : diagnostic duration	103	128	201	0.011	0.012	0.002
Diabetes	diabetes-related eye disease : diagnostic age	104	166	187	0.011	0.007	0.003
Body composition and body morphology	Arm fat percentage (right)	105	261	150	0.011	80518	0.005
Body injuries and illnesses	Non-cancer illness: diagnostic duration (seventh time)	106	177	97	0.010	0.006	0.012
Body injuries and illnesses	Asthma : diagnostic duration	107	106	122	0.010	0.015	0.009

Eye/Vision impairment	Loss of vision due to injury or trauma: diagnostic duration	108	176	165	0.010	0.006	0.004
Body composition and body morphology	Leg fat percentage (left)	109	257	217	0.010	0.0001	0.001
Body injuries and illnesses	Non-cancer illness: diagnostic age (fifth time)	110	123	149	0.010	0.013	0.005
Baseline characteristics	Standard PRS for age at menopause (AAM)	111	137	138	0.009	0.010	0.007
Body injuries and illnesses	Emphysema/Chronic bronchitis : diagnostic duration	112	206	197	0.009	0.003	0.002
Body composition and body morphology	Impedance of whole body	113	154	113	0.009	0.008	0.010
Body injuries and illnesses	Non-cancer illness: diagnostic age (seventh time)	114	218	131	0.009	0.002	0.008
Body injuries and illnesses	Lung cancer (not mesothelioma) : diagnostic duration	115	241	250	0.009	0.001	0.0004
Body injuries and illnesses	Operation : age when took place (third time)	116	233	58	0.009	0.001	0.021
Body composition and body morphology	Leg fat mass (right)	117	196	141	0.009	0.004	0.006
Eye/Vision impairment	Macular degeneration : diagnostic duration	118	199	247	0.009	0.004	0.0004
Body injuries and illnesses	Emphysema/Chronic bronchitis : diagnostic age	119	189	184	0.009	0.004	0.003
Body injuries and illnesses	Operation : duration of taking place (sixth time)	120	256	258	0.009	0.000	0.0001
Body composition and body morphology	Weight	121	179	160	0.009	0.006	0.004
Mental health and socio-affective factors	General happiness with own health	122	33	57	0.009	0.033	0.021
Body injuries and illnesses	Cancer : diagnostic duration (second time)	123	184	229	0.009	0.005	0.001
Body injuries and illnesses	Chronic Obstructive Pulmonary Disease : diagnostic age	124	203	213	0.008	0.003	0.001
Body composition and body morphology	Leg fat percentage (right)	125	223	202	0.008	0.002	0.002
Body composition and body morphology	Trunk fat percentage	126	110	151	0.008	0.015	0.005
Body composition and body morphology	Arm fat percentage (left)	127	217	181	0.008	0.002	0.004
Body composition and body morphology	Leg predicted mass (left)	128	165	177	0.008	0.007	0.004
Body injuries and illnesses	Non-cancer illness: diagnostic age (sixth time)	129	127	238	0.008	0.013	0.0007
Body composition and body morphology	Leg predicted mass (right)	130	152	174	0.008	0.008	0.004
Body composition and body morphology	Leg fat-free mass (left)	131	167	175	0.007	0.007	0.004
Body composition and body morphology	Basal metabolic rate	132	201	193	0.007	0.004	0.003
Body injuries and illnesses	Non-cancer illness: diagnostic age (eighth time)	133	232	162	0.007	0.001	0.004
Cardiovascular health	Heart attack : diagnostic age	134	160	188	0.007	0.008	0.003
Mental health and socio-affective factors	Belittlement by partner or ex-partner as an adult	135	126	105	0.007	0.013	0.011
Diet and alcohol	Exclude eggs, dairy, wheat, sugar from diet	136	114	25	0.007	0.014	0.030
Cardiovascular health	Angina : diagnostic age	137	221	190	0.007	0.002	0.003
Body composition and body morphology	Arm fat-free mass (right)	138	234	220	0.007	0.001	0.001
Cardiovascular health	Pulmonary embolism (blood clot in lung) : diagnostic age	139	253	237	0.007	0.000	0.0008
Body composition and body morphology	Arm predicted mass (right)	140	259	239	0.007	51853	0.0007
Body composition and body morphology	Body mass index (BMI)	141	173	252	0.007	0.007	0.0002
Body composition and body morphology	Leg fat-free mass (right)	142	149	173	0.007	0.008	0.004
Body composition and body morphology	Arm fat mass (left)	143	250	186	0.007	0.0006	0.003
Body injuries and illnesses	Lung cancer (not mesothelioma) : diagnostic age	144	240	249	0.006	0.001	0.0004

Body composition and body morphology	Leg fat mass (left)	145	239	148	0.006	0.001	0.005
Body injuries and illnesses	Operation : age when took place (fourth time)	146	171	125	0.006	0.007	0.009
Body injuries and illnesses	Cancer : diagnostic age (second time)	147	182	261	0.006	0.005	0.0
Body composition and body morphology	Arm predicted mass (left)	148	255	256	0.006	0.0002	0.0001
Body composition and body morphology	Trunk predicted mass	149	242	257	0.006	0.001	0.0001
Body composition and body morphology	Arm fat-free mass (left)	150	254	233	0.006	0.0002	0.0009
Diet and alcohol	Cheese intake	151	29	34	0.006	0.036	0.027
Body composition and body morphology	Weight change in last year	152	5	10	0.006	0.069	0.049
Body injuries and illnesses	Pain type(s) experienced in last month	153	68	107	0.006	0.023	0.011
Cardiovascular health	Vascular/heart problems	154	17	21	0.005	0.045	0.033
Body composition and body morphology	Trunk fat mass	155	102	80	0.005	0.016	0.016
Mental health and socio-affective factors	Felt loved as a child	156	72	140	0.005	0.022	0.006
Body composition and body morphology	Trunk fat-free mass	157	247	253	0.005	0.0008	0.0002
Body composition and body morphology	Arm fat mass (right)	158	246	196	0.005	0.0009	0.002
Body composition and body morphology	Body fat percentage	159	187	244	0.005	0.005	0.0005
Cardiovascular health	Heart attack : diagnostic duration	160	235	180	0.005	0.001	0.004
Diet and alcohol	Poultry intake	161	175	109	0.005	0.006	0.011
Body composition and body morphology	Whole body fat-free mass	162	222	216	0.005	0.002	0.001
Cardiovascular health	Pulmonary embolism (blood clot in lung) : diagnostic duration	163	249	215	0.005	0.000	0.001
Mental health and socio-affective factors	Confiding Relationships in Adulthood	164	70	15	0.005	0.022	0.040
Early life factors	Comparative body size at age 10	165	87	19	0.005	0.019	0.036
Body injuries and illnesses	Wheeze or whistling in the chest in last year	166	214	54	0.005	0.002	0.022
Mental health and socio-affective factors	Able to pay rent/mortgage as an adult	167	134	110	0.005	0.011	0.011
Mental health and socio-affective factors	Long-term worry and tension	168	204	53	0.005	0.003	0.022
Sociodemographics	Qualifications	169	116	158	0.005	0.014	0.004
Mental health and socio-affective factors	General happiness	170	25	24	0.005	0.040	0.030
Mental health and socio-affective factors	Work/job satisfaction	171	55	73	0.004	0.026	0.018
Body injuries and illnesses	Mineral and other dietary supplements	172	115	35	0.004	0.014	0.026
Body injuries and illnesses	Medication for pain relief, constipation, heartburn	173	248	96	0.004	0.0008	0.013
Sociodemographics	Years of education	174	190	120	0.004	0.004	0.010
Mental health and socio-affective factors	Physically abused by family as a child	175	103	66	0.004	0.016	0.019
Body composition and body morphology	Whole body fat mass	176	169	152	0.004	0.007	0.005
Body injuries and illnesses	Overall health rating	177	92	94	0.004	0.018	0.013
Diet and alcohol	Pork intake	178	74	38	0.004	0.022	0.026
Diet and alcohol	Vitamin and mineral supplements	179	183	119	0.003	0.005	0.010
Body composition and body morphology	Whole body water mass	180	227	211	0.003	0.002	0.001
Mental health and socio-affective factors	Felt hated by family member as a child	181	95	121	0.003	0.017	0.009

Diet and alcohol	Beef intake	182	122	91	0.003	0.013	0.014
Mental health and socio-affective factors	Disturbing thoughts of past stress lately	183	77	170	0.003	0.021	0.004
Diet and alcohol	Oily fish intake	184	19	52	0.003	0.044	0.022
Mental health and socio-affective factors	Worried more than others in similar situations	185	13	30	0.003	0.053	0.027
Diet and alcohol	Major dietary changes in the last 5 years	186	224	114	0.003	0.002	0.010
Diet and alcohol	Processed meat intake	187	161	74	0.003	0.008	0.018
Mental health and socio-affective factors	Belief that own life is meaningful	188	132	102	0.003	0.011	0.011
Mental health and socio-affective factors	Thought life was not worth living	189	59	78	0.003	0.025	0.016
Mental health and socio-affective factors	Family relationship satisfaction	190	18	101	0.003	0.045	0.012
Body injuries and illnesses	Life-threatening illness	191	22	23	0.003	0.042	0.030
Diet and alcohol	Milk type used	192	118	45	0.003	0.013	0.023
Diet and alcohol	Variation in diet	193	85	37	0.003	0.020	0.026
Diet and alcohol	Non-oily fish intake	194	120	31	0.003	0.013	0.027
Diet and alcohol	Lamb/mutton intake	195	138	179	0.003	0.010	0.004
Diet and alcohol	Hot drink temperature	196	36	27	0.003	0.033	0.030
Mental health and socio-affective factors	Physical violence from ex-/partner	197	94	103	0.003	0.017	0.011
Mental health and socio-affective factors	Addiction to any substance or behaviour	198	136	43	0.003	0.011	0.023
Mental health and socio-affective factors	Sleep Disruptions: Difficulty Falling or Staying Asleep	199	251	178	0.003	0.000	0.004
Mental health and socio-affective factors	Financial situation satisfaction	200	58	136	0.002	0.026	0.007
Mental health and socio-affective factors	Friendships satisfaction	201	157	167	0.002	0.008	0.004
Mental health and socio-affective factors	Difficulty stopping worry lately	202	113	77	0.002	0.014	0.017
Diet and alcohol	Salt added to food	203	144	245	0.002	0.009	0.0004
Cardiovascular health	Blood Clot, DVT, and Respiratory Conditions	204	140	69	0.002	0.010	0.018
Body injuries and illnesses	Other prescription medications	205	163	142	0.002	0.008	0.006
Mental health and socio-affective factors	Difficulty relaxing lately	206	28	41	0.002	0.037	0.025
Cardiovascular health	Smoking status	207	26	13	0.002	0.038	0.043
Mental health and socio-affective factors	Mental distress affecting daily activities	208	194	192	0.002	0.004	0.003
Mental health and socio-affective factors	Feelings of tiredness/low energy lately	209	142	157	0.002	0.010	0.004
Body injuries and illnesses	Falls in the last year	210	60	32	0.002	0.024	0.027
Mental health and socio-affective factors	Felt upset by reminders of past stress	211	99	93	0.002	0.017	0.013
Early life factors	Comparative height size at age 10	212	56	168	0.002	0.026	0.004
Diet and alcohol	Bread type	213	73	159	0.002	0.022	0.004
Mental health and socio-affective factors	Trouble focusing on tasks lately	214	131	130	0.002	0.012	0.008
Diet and alcohol	Spread type	215	191	143	0.002	0.004	0.006
Body injuries and illnesses	Long-standing illness, disability or infirmity	216	100	254	0.001	0.016	0.0001
Early life factors	Maternal smoking around birth	217	216	207	0.001	0.002	0.002
Mental health and socio-affective factors	Sought help for mental distress	218	101	124	0.001	0.016	0.009
Mental health and socio-affective factors	Childhood Health: The Role of a Supportive Caregiver	219	238	118	0.001	0.001	0.010

Lifestyle and environment	Home area population density - urban or rural	220	219	226	0.001	0.002	0.001
Mental health and socio-affective factors	Long-term loss of interest in activities	221	45	224	0.001	0.030	0.001
Mental health and socio-affective factors	Victim of physically violent crime	222	180	48	0.001	0.006	0.023
Sociodemographics	Encoded anonymised participant ID	223	129	198	0.001	0.012	0.002
Mental health and socio-affective factors	Witnessed sudden violent death	224	109	223	0.001	0.015	0.001
Lifestyle and environment	Noisy workplace	225	37	139	0.001	0.033	0.006
Mental health and socio-affective factors	Easily annoyed or irritable lately	226	31	209	0.001	0.036	0.001
Mental health and socio-affective factors	Feelings of depression lately	227	143	134	0.001	0.009	0.007
Mental health and socio-affective factors	Excessive worrying lately	228	153	231	0.001	0.008	0.001
Mental health and socio-affective factors	Period of extreme irritability	229	80	163	0.001	0.021	0.004
Mental health and socio-affective factors	Cannabis usage	230	170	79	0.001	0.007	0.016
Diabetes	Diabetes : diagnosis	231	51	44	0.001	0.028	0.023
Mental health and socio-affective factors	Feelings of anxiety/nervousness lately	232	244	135	0.001	0.001	0.007
Mental health and socio-affective factors	Feelings of inadequacy lately	233	65	68	0.001	0.024	0.018
Body injuries and illnesses	Other serious medical conditions and disabilities	234	84	129	0.001	0.020	0.009
Mental health and socio-affective factors	Contemplated self-harm	235	125	183	0.001	0.013	0.003
Mental health and socio-affective factors	Activities Avoided Due to Recent Stress	236	197	194	0.001	0.004	0.003
Lifestyle and environment	Facial ageing	237	39	166	0.001	0.033	0.004
Mental health and socio-affective factors	Serious Life-Threatening Accident	238	38	89	0.001	0.033	0.014
Mental health and socio-affective factors	Poor appetite and overeating lately	239	174	137	0.001	0.006	0.007
Diet and alcohol	Injury Caused by alcohol consumption	240	90	115	0.001	0.018	0.010
Mental health and socio-affective factors	Victim of sexual assault	241	48	242	0.0009	0.029	0.0006
Lifestyle and environment	Loud music exposure frequency	242	30	85	0.0009	0.036	0.015
Mental health and socio-affective factors	Sexually molested as a child	243	64	63	0.0007	0.024	0.020
Mental health and socio-affective factors	Diminished pleasure in activities lately	244	141	145	0.0006	0.010	0.006
Body injuries and illnesses	Chest pain or discomfort	245	156	64	0.0006	0.008	0.020
Mental health and socio-affective factors	Feelings of restlessness lately	246	43	86	0.0006	0.030	0.015
Mental health and socio-affective factors	Long-lasting sadness or depression	247	202	222	0.0006	0.003	0.001
Mental health and socio-affective factors	Feelings of foreboding lately	248	158	147	0.0006	0.008	0.006
Mental health and socio-affective factors	Period of mania / excitability	249	145	133	0.0005	0.009	0.007
Early life factors	Handedness (chirality/laterality)	250	198	176	0.0003	0.004	0.004
Mental health and socio-affective factors	Non-consensual sexual Interference by ex-/partners	251	81	108	0.0002	0.020	0.011
Mental health and socio-affective factors	Ever self-harmed	252	205	88	0.0001	0.003	0.014
Mental health and socio-affective factors	Changes in speed/amount of moving or speaking lately	253	192	189	0.0001	0.004	0.003
Mental health and socio-affective factors	Having thoughts of self-harm lately	254	200	236	0.0001	0.004	0.0009
Mental health and socio-affective factors	Belief in a personal conspiracy	255	260	235	0.0	0.0	0.0009
Mental health and socio-affective factors	Belief in unreal communications	256	213	248	0.0	0.002	0.0004

Mental health and socio-affective factors	heard an un-real voice	257	164	99	0.0	0.007	0.012
Mental health and socio-affective factors	Combat/War-Zone Involvement	258	178	144	0.0	0.006	0.006
Mental health and socio-affective factors	seen an un-real vision	259	151	185	0.0	0.008	0.003
Body injuries and illnesses	Cancer : diagnosis	260	252	161	0.0	0.0006	0.004
Sociodemographics	Race	261	195	199	0.0	0.004	0.002

Supplementary Table 7. Feature importance ranking of all exposome variables in different subsets: Feature importance ranking of all exposome variables in different subsets including main subset (*n* = 3706), replication subset (*n* = 4202), and variable-restricted subset (*n* = 7736).

Category	Title	Ranking - Main subset	Ranking - Replication subset	Ranking - variables-restricted subset	Main Subset (mean absolute SHAP value)	Replication Subset (mean absolute SHAP value)	variables-restricted Subset (mean absolute SHAP value)
Cardiovascular health	High blood pressure : diagnostic age	1	1	6	0.222	0.310	0.162
Eye/Vision impairment	Loss of vision due to injury or trauma: diagnostic age	2	39	68	0.194	0.024	0.015
Body composition and body morphology	Sitting height	3	5	5	0.177	0.148	0.178
Diabetes	Diabetes disease : diagnostic duration	4	9	3	0.161	0.075	0.188
Cardiovascular health	Smoking : duration	5	2	2	0.140	0.270	0.203
Cardiovascular health	Diastolic blood pressure, automated reading	6	4	4	0.110	0.152	0.179
Cardiovascular health	Smoking : age when started	7	15	56	0.105	0.045	0.017
Diet and alcohol	Coffee intake	8	6	8	0.103	0.101	0.105
Body injuries and illnesses	Cancer : diagnostic duration (first time)	9	3	20	0.101	0.162	0.035
Cardiovascular health	Smoking : age when stopped	10	8	13	0.090	0.091	0.063
Diet and alcohol	Dried fruit intake	11	10	11	0.080	0.069	0.072
Eye/Vision impairment	Glaucoma : diagnostic age	12	21	123	0.079	0.038	0.007
Diet and alcohol	Cereal intake	13	7	9	0.069	0.094	0.095
Diabetes	Diabetes-related eye disease : diagnostic duration	14	12	102	0.063	0.055	0.010
Bone health	Heel bone mineral density (BMD) T-score, automated (right)	15			0.061		
Bone health	Heel bone mineral density (BMD) T-score, automated (left)	16			0.056		
Diabetes	Diabetes disease : diagnostic age	17	20	12	0.053	0.038	0.072
Mental health and socio-affective factors	Unusual or psychotic experience : diagnostic age	18	14	62	0.052	0.046	0.016
Body injuries and illnesses	Operation : age when took place (first time)	19	28	30	0.049	0.028	0.027
Diet and alcohol	Alcohol intake	20	25		0.046	0.033	
Cardiovascular health	High blood pressure : diagnostic duration	21	17	10	0.043	0.043	0.083
Body injuries and illnesses	Operation : age when took place (second time)	22	26	14	0.043	0.033	0.051
Cardiovascular health	Pulse rate	23	18	19	0.040	0.040	0.035
Diet and alcohol	Cooked vegetable intake	24	38	43	0.040	0.024	0.019
Body composition and body morphology	Hip circumference	25	32	33	0.035	0.026	0.025
Body injuries and illnesses	Cancer : diagnostic age (first time)	26	23	50	0.032	0.035	0.018
Body injuries and illnesses	Non-cancer illness: diagnostic age (first time)	27	49	51	0.032	0.020	0.018

Body injuries and illnesses	Non-cancer illness: diagnostic duration (first time)	28	30	63	0.030	0.027	0.016
Cardiovascular health	Smoking : age when started again (current smokers)	29	24	59	0.029	0.035	0.016
Body composition and body morphology	Hand grip strength (left)	30	22	15	0.029	0.036	0.045
Diet and alcohol	Bread intake	31	43	31	0.029	0.022	0.027
Body injuries and illnesses	Non-cancer illnesses : self-reported number	32	42	23	0.029	0.022	0.031
Body injuries and illnesses	Cancers count : self-reported number	33	77	117	0.029	0.013	0.008
Body composition and body morphology	Forced vital capacity (FVC)	34	33	24	0.028	0.025	0.030
Diet and alcohol	Salad / raw vegetable intake	35	44	55	0.028	0.022	0.017
Body injuries and illnesses	Operations count : self-reported number	36	35	36	0.027	0.025	0.022
Diet and alcohol	Fresh fruit intake	37	40	35	0.027	0.023	0.023
Mental health and socio-affective factors	Depression : age at last episode	38	34	96	0.026	0.025	0.011
Mental health and socio-affective factors	Unusual or psychotic experience : diagnostic duration	39	16	54	0.026	0.043	0.017
Lifestyle and environment	Duration of moderate activity	40	29	28	0.025	0.028	0.028
Body injuries and illnesses	Chronic bronchitis : diagnostic age	41	37	42	0.025	0.024	0.019
Mental health and socio-affective factors	Cannabis : age when last took	42	36	38	0.024	0.024	0.021
Eye/Vision impairment	Macular degeneration : diagnostic age	43	67	82	0.024	0.015	0.012
Cardiovascular health	Angina : diagnostic duration	44	104	145	0.024	0.009	0.006
Body injuries and illnesses	Operation : duration of taking place (first time)	45	56	47	0.023	0.017	0.018
Diet and alcohol	Water intake	46	27	18	0.022	0.028	0.035
Early life factors	Birth weight	47	61	34	0.022	0.016	0.024
Diet and alcohol	Alcohol intake frequency	48	54	17	0.022	0.018	0.036
Body composition and body morphology	Hand grip strength (right)	49	47	25	0.021	0.021	0.029
Cardiovascular health	Systolic blood pressure, automated reading	50	50	49	0.021	0.020	0.018
Cardiovascular health	Pulse wave Arterial Stiffness index	51	76	52	0.020	0.013	0.018
Body composition and body morphology	Forced expiratory volume in 1-second (FEV1)	52	53	26	0.020	0.019	0.029
Eye/Vision impairment	Wearing glasses or lenses : age when started	53	41	41	0.020	0.023	0.019
Body injuries and illnesses	Emphysema : diagnostic age	54	55	93	0.020	0.018	0.011
Body injuries and illnesses	Non-cancer illness: diagnostic duration (second time)	55	48	27	0.020	0.020	0.028
Body injuries and illnesses	Non-cancer illness: diagnostic duration (third time)	56	19	16	0.020	0.039	0.043
Body composition and body morphology	Impedance of arm (left)	57	60	67	0.019	0.016	0.015
Body composition and body morphology	Waist circumference	58	45	21	0.018	0.021	0.034

Body composition and body morphology	Impedance of leg (right)	59	100	77	0.018	0.010	0.013
Body injuries and illnesses	Non-cancer illness: diagnostic duration (eighth time)	60	13	149	0.018	0.048	0.005
Body composition and body morphology	Peak expiratory flow (PEF)	61	51	22	0.017	0.020	0.031
Diet and alcohol	Known person advocated alcohol reduction	62	74		0.017	0.013	
Diet and alcohol	Tea intake	63	59	37	0.017	0.016	0.022
Body injuries and illnesses	Asthma : diagnostic age by doctor	64	46	95	0.017	0.021	0.011
Eye/Vision impairment	Cataract : diagnostic age	65	91	89	0.016	0.010	0.011
Body injuries and illnesses	Non-cancer illness: diagnostic age (third time)	66	57	101	0.016	0.017	0.010
Cardiovascular health	Pulse rate, automated reading	67	83	57	0.016	0.012	0.017
Cardiovascular health	Stroke : diagnostic duration	68	95	121	0.016	0.010	0.008
Body injuries and illnesses	Operation : duration of taking place (fourth time)	69	113	94	0.016	0.008	0.011
Body injuries and illnesses	Asthma : diagnostic age	70	124	48	0.016	0.007	0.018
Body injuries and illnesses	Non-cancer illness: diagnostic age (fourth time)	71	98	76	0.016	0.010	0.013
Body injuries and illnesses	Sarcoidosis : diagnostic duration	72	101	112	0.016	0.010	0.009
Eye/Vision impairment	Wearing glasses or lenses : duration from when started	73	69	66	0.016	0.015	0.015
Body injuries and illnesses	Operation : duration of taking place (second time)	74	64	29	0.016	0.015	0.027
Mental health and socio-affective factors	Depression : duration from first episode	75	68	79	0.016	0.015	0.013
Body composition and body morphology	Impedance of arm (right)	76	73	70	0.015	0.013	0.014
Body injuries and illnesses	Emphysema : diagnostic duration	77	72	73	0.015	0.013	0.014
Body injuries and illnesses	Chronic bronchitis : diagnostic age	78	78	107	0.015	0.013	0.009
Body injuries and illnesses	Sarcoidosis : diagnostic age	79	89	1	0.015	0.011	0.357
Mental health and socio-affective factors	Depression : duration from last episode	80	52	71	0.014	0.019	0.014
Cardiovascular health	Stroke : diagnostic age	81	119	139	0.014	0.007	0.006
Eye/Vision impairment	Standard PRS for age-related macular degeneration (AMD)	82	85	110	0.014	0.011	0.009
Body injuries and illnesses	Hay fever, rhinitis or eczema : diagnostic age	83	97	60	0.014	0.010	0.016
Body injuries and illnesses	Tuberculosis : diagnostic age	84	144	80	0.014	0.005	0.013
Mental health and socio-affective factors	Depression : age at first episode	85	71	69	0.014	0.014	0.015
Body injuries and illnesses	Non-cancer illness: diagnostic duration (fourth time)	86	80	32	0.014	0.013	0.026
Body injuries and illnesses	Hay fever, rhinitis or eczema : diagnostic duration	87	63	44	0.013	0.015	0.019
Body injuries and illnesses	Non-cancer illness: diagnostic age (second time)	88	70	61	0.013	0.014	0.016

Body injuries and illnesses	Operation : age when took place (fifth time)	89	82	86	0.013	0.012	0.011
Eye/Vision impairment	Glaucoma : diagnostic duration	90	116	115	0.013	0.008	0.008
Mental health and socio-affective factors	Cannabis : duration from when last took	91	106	75	0.013	0.009	0.014
Body injuries and illnesses	Operation : age when took place (sixth time)	92	92	113	0.013	0.010	0.008
Body injuries and illnesses	Non-cancer illness: diagnostic duration (fifth time)	93	123	91	0.013	0.007	0.011
Body injuries and illnesses	Chronic Obstructive Pulmonary Disease : diagnostic duration	94	58	74	0.012	0.016	0.014
Body injuries and illnesses	Tuberculosis : diagnostic duration	95	99	72	0.012	0.010	0.014
Body injuries and illnesses	Asthma : diagnostic (by doctor) duration	96	112	40	0.012	0.008	0.020
Body injuries and illnesses	Hayfever or allergic rhinitis : diagnostic age	97	62	118	0.012	0.016	0.008
Body injuries and illnesses	Operation : duration of taking place (fifth time)	98	81	81	0.012	0.012	0.012
Body injuries and illnesses	Operation : duration of taking place (third time)	99	84	65	0.011	0.012	0.015
Body injuries and illnesses	Non-cancer illness: diagnostic duration (sixth time)	100	127	129	0.011	0.007	0.006
Eye/Vision impairment	Cataract : diagnostic duration	101	75	127	0.011	0.013	0.007
Body composition and body morphology	Impedance of leg (left)	102	87	92	0.011	0.011	0.011
Body injuries and illnesses	Hayfever or allergic rhinitis : diagnostic duration	103	109	88	0.011	0.009	0.011
Diabetes	diabetes-related eye disease : diagnostic age	104	103	134	0.011	0.009	0.006
Body composition and body morphology	Arm fat percentage (right)	105	120	106	0.011	0.007	0.009
Body injuries and illnesses	Non-cancer illness: diagnostic duration (seventh time)	106	102	141	0.010	0.009	0.006
Body injuries and illnesses	Asthma : diagnostic duration	107	66	45	0.010	0.015	0.019
Eye/Vision impairment	Loss of vision due to injury or trauma: diagnostic duration	108	31	46	0.010	0.027	0.019
Body composition and body morphology	Leg fat percentage (left)	109	88	120	0.010	0.011	0.008
Body injuries and illnesses	Non-cancer illness: diagnostic age (fifth time)	110	65	78	0.010	0.015	0.013
Baseline characteristics	Standard PRS for age at menopause (AAM)	111	121	114	0.009	0.007	0.008
Body injuries and illnesses	Emphysema/Chronic bronchitis : diagnostic duration	112	96	97	0.009	0.010	0.010
Body composition and body morphology	Impedance of whole body	113	108	99	0.009	0.009	0.010
Body injuries and illnesses	Non-cancer illness: diagnostic age (seventh time)	114	94	133	0.009	0.010	0.006
Body injuries and illnesses	Lung cancer (not mesothelioma) : diagnostic duration	115	148	104	0.009	0.005	0.009
Body injuries and illnesses	Operation : age when took place (third time)	116	130	85	0.009	0.007	0.011
Body composition and body morphology	Leg fat mass (right)	117	158	131	0.009	0.004	0.006
Eye/Vision impairment	Macular degeneration : diagnostic duration	118	11	128	0.009	0.062	0.007

Body injuries and illnesses	Emphysema/Chronic bronchitis : diagnostic age	119	131	58	0.009	0.006	0.016
Body injuries and illnesses	Operation : duration of taking place (sixth time)	120	117	119	0.009	0.007	0.008
Body composition and body morphology	Weight	121	114	161	0.009	0.008	0.004
Mental health and socio-affective factors	General happiness with own health	122	86		0.009	0.011	
Body injuries and illnesses	Cancer : diagnostic duration (second time)	123	143	53	0.009	0.005	0.018
Body injuries and illnesses	Chronic Obstructive Pulmonary Disease : diagnostic age	124	79	98	0.008	0.013	0.010
Body composition and body morphology	Leg fat percentage (right)	125	105	83	0.008	0.009	0.012
Body composition and body morphology	Trunk fat percentage	126	90	87	0.008	0.010	0.011
Body composition and body morphology	Arm fat percentage (left)	127	122	105	0.008	0.007	0.009
Body composition and body morphology	Leg predicted mass (left)	128	118	160	0.008	0.007	0.004
Body injuries and illnesses	Non-cancer illness: diagnostic age (sixth time)	129	115	90	0.008	0.008	0.011
Body composition and body morphology	Leg predicted mass (right)	130	129	152	0.008	0.007	0.005
Body composition and body morphology	Leg fat-free mass (left)	131	147	157	0.007	0.005	0.005
Body composition and body morphology	Basal metabolic rate	132	163	125	0.007	0.004	0.007
Body injuries and illnesses	Non-cancer illness: diagnostic age (eighth time)	133	93	144	0.007	0.010	0.006
Cardiovascular health	Heart attack : diagnostic age	134	171	146	0.007	0.004	0.006
Mental health and socio-affective factors	Belittlement by partner or ex-partner as an adult	135	136		0.007	0.006	
Diet and alcohol	Exclude eggs, dairy, wheat, sugar from diet	136	152	162	0.007	0.005	0.004
Cardiovascular health	Angina : diagnostic age	137	169	108	0.007	0.004	0.009
Body composition and body morphology	Arm fat-free mass (right)	138	111	103	0.007	0.009	0.010
Cardiovascular health	Pulmonary embolism (blood clot in lung) : diagnostic age	139	110	7	0.007	0.009	0.122
Body composition and body morphology	Arm predicted mass (right)	140	167	132	0.007	0.004	0.006
Body composition and body morphology	Body mass index (BMI)	141	145	116	0.007	0.005	0.008
Body composition and body morphology	Leg fat-free mass (right)	142	139	164	0.007	0.006	0.004
Body composition and body morphology	Arm fat mass (left)	143	141	124	0.007	0.005	0.007
Body injuries and illnesses	Lung cancer (not mesothelioma) : diagnostic age	144	126	64	0.006	0.007	0.015
Body composition and body morphology	Leg fat mass (left)	145	133	84	0.006	0.006	0.012

Body injuries and illnesses	Operation : age when took place (fourth time)	146	138	148	0.006	0.006	0.005
Body injuries and illnesses	Cancer : diagnostic age (second time)	147	107	138	0.006	0.009	0.006
Body composition and body morphology	Arm predicted mass (left)	148	128	126	0.006	0.007	0.007
Body composition and body morphology	Trunk predicted mass	149	153	158	0.006	0.005	0.004
Body composition and body morphology	Arm fat-free mass (left)	150	132	136	0.006	0.006	0.006
Diet and alcohol	Cheese intake	151	177	178	0.006	0.003	0.003
Body composition and body morphology	Weight change in last year	152	135	122	0.006	0.006	0.007
Body injuries and illnesses	Pain type(s) experienced in last month	153	146	100	0.006	0.005	0.010
Cardiovascular health	Vascular/heart problems	154	140	130	0.005	0.006	0.006
Body composition and body morphology	Trunk fat mass	155	137	135	0.005	0.006	0.006
Mental health and socio-affective factors	Felt loved as a child	156	154		0.005	0.005	
Body composition and body morphology	Trunk fat-free mass	157	168	163	0.005	0.004	0.004
Body composition and body morphology	Arm fat mass (right)	158	150	155	0.005	0.005	0.005
Body composition and body morphology	Body fat percentage	159	134	111	0.005	0.006	0.009
Cardiovascular health	Heart attack : diagnostic duration	160	161	109	0.005	0.004	0.009
Diet and alcohol	Poultry intake	161	155	154	0.005	0.005	0.005
Body composition and body morphology	Whole body fat-free mass	162	181	167	0.005	0.003	0.003
Cardiovascular health	Pulmonary embolism (blood clot in lung) : diagnostic duration	163	125	39	0.005	0.007	0.020
Mental health and socio-affective factors	Confiding Relationships in Adulthood	164	173		0.005	0.003	
Early life factors	Comparative body size at age 10	165	185	171	0.005	0.003	0.003
Body injuries and illnesses	Wheeze or whistling in the chest in last year	166	203	195	0.005	0.002	0.001
Mental health and socio-affective factors	Able to pay rent/mortgage as an adult	167	151		0.005	0.005	
Mental health and socio-affective factors	Long-term worry and tension	168	164		0.005	0.004	
Sociodemographics	Qualifications	169	176	166	0.005	0.003	0.004
Mental health and socio-affective factors	General happiness	170	180		0.005	0.003	
Mental health and socio-affective factors	Work/job satisfaction	171	142	153	0.004	0.005	0.005
Body injuries and illnesses	Mineral and other dietary supplements	172	184	184	0.004	0.003	0.002
Body injuries and illnesses	Medication for pain relief, constipation, heartburn	173	182	142	0.004	0.003	0.006
Sociodemographics	Years of education	174	196	188	0.004	0.002	0.002

Mental health and socio-affective factors	Physically abused by family as a child	175	219		0.004	0.001	
Body composition and body morphology	Whole body fat mass	176	160	168	0.004	0.004	0.003
Body injuries and illnesses	Overall health rating	177	178	173	0.004	0.003	0.003
Diet and alcohol	Pork intake	178	187	156	0.004	0.003	0.005
Diet and alcohol	Vitamin and mineral supplements	179	206	151	0.003	0.002	0.005
Body composition and body morphology	Whole body water mass	180	156	169	0.003	0.004	0.003
Mental health and socio-affective factors	Felt hated by family member as a child	181	232		0.003	0.001	
Diet and alcohol	Beef intake	182	183	150	0.003	0.003	0.005
Mental health and socio-affective factors	Disturbing thoughts of past stress lately	183	166		0.003	0.004	
Diet and alcohol	Oily fish intake	184	186	147	0.003	0.003	0.005
Mental health and socio-affective factors	Worried more than others in similar situations	185	188		0.003	0.003	
Diet and alcohol	Major dietary changes in the last 5 years	186	199	185	0.003	0.002	0.002
Diet and alcohol	Processed meat intake	187	149	137	0.003	0.005	0.006
Mental health and socio-affective factors	Belief that own life is meaningful	188	157		0.003	0.004	
Mental health and socio-affective factors	Thought life was not worth living	189	195		0.003	0.002	
Mental health and socio-affective factors	Family relationship satisfaction	190	172	140	0.003	0.004	0.006
Body injuries and illnesses	Life-threatening illness	191	230		0.003	0.001	
Diet and alcohol	Milk type used	192	198	175	0.003	0.002	0.003
Diet and alcohol	Variation in diet	193	197	181	0.003	0.002	0.002
Diet and alcohol	Non-oily fish intake	194	192	177	0.003	0.002	0.003
Diet and alcohol	Lamb/mutton intake	195	190	187	0.003	0.002	0.002
Diet and alcohol	Hot drink temperature	196	200	174	0.003	0.002	0.003
Mental health and socio-affective factors	Physical violence from ex-/partner	197	216		0.003	0.001	
Mental health and socio-affective factors	Addiction to any substance or behaviour	198	189		0.003	0.002	
Mental health and socio-affective factors	Sleep Disruptions: Difficulty Falling or Staying Asleep	199	191		0.003	0.002	
Mental health and socio-affective factors	Financial situation satisfaction	200	179	170	0.002	0.003	0.003
Mental health and socio-affective factors	Friendships satisfaction	201	208	179	0.002	0.002	0.003
Mental health and socio-affective factors	Difficulty stopping worry lately	202	175		0.002	0.003	
Diet and alcohol	Salt added to food	203	204	159	0.002	0.002	0.004
Cardiovascular health	Blood Clot, DVT, and Respiratory Conditions	204	170	189	0.002	0.004	0.002

Body injuries and illnesses	Other prescription medications	205	214	165	0.002	0.001	0.004
Mental health and socio-affective factors	Difficulty relaxing lately	206	174		0.002	0.003	
Cardiovascular health	Smoking status	207	159	143	0.002	0.004	0.006
Mental health and socio-affective factors	Mental distress affecting daily activities	208	226		0.002	0.001	
Mental health and socio-affective factors	Feelings of tiredness/low energy lately	209	162		0.002	0.004	
Body injuries and illnesses	Falls in the last year	210	239	193	0.002	0.000	0.001
Mental health and socio-affective factors	Felt upset by reminders of past stress	211	165		0.002	0.004	
Early life factors	Comparative height size at age 10	212	205	172	0.002	0.002	0.003
Diet and alcohol	Bread type	213	202	180	0.002	0.002	0.003
Mental health and socio-affective factors	Trouble focusing on tasks lately	214	225		0.002	0.001	
Diet and alcohol	Spread type	215	209	176	0.002	0.002	0.003
Body injuries and illnesses	Long-standing illness, disability or infirmity	216	207	186	0.001	0.002	0.002
Early life factors	Maternal smoking around birth	217	212	190	0.001	0.001	0.001
Mental health and socio-affective factors	Sought help for mental distress	218	229		0.001	0.001	
Mental health and socio-affective factors	Childhood Health: The Role of a Supportive Caregiver	219	224		0.001	0.001	
Lifestyle and environment	Home area population density - urban or rural	220	227	183	0.001	0.001	0.002
Mental health and socio-affective factors	Long-term loss of interest in activities	221	215		0.001	0.001	
Mental health and socio-affective factors	Victim of physically violent crime	222	221		0.001	0.001	
Sociodemographics	Encoded anonymised participant ID	223	211	194	0.001	0.001	0.001
Mental health and socio-affective factors	Witnessed sudden violent death	224	223		0.001	0.001	
Lifestyle and environment	Noisy workplace	225	236	191	0.001	0.001	0.001
Mental health and socio-affective factors	Easily annoyed or irritable lately	226	193		0.001	0.002	
Mental health and socio-affective factors	Feelings of depression lately	227	217		0.001	0.001	
Mental health and socio-affective factors	Excessive worrying lately	228	233		0.001	0.001	
Mental health and socio-affective factors	Period of extreme irritability	229	235		0.001	0.001	
Mental health and socio-affective factors	Cannabis usage	230	210		0.001	0.002	
Diabetes	Diabetes : diagnosis	231	201	198	0.001	0.002	0.001
Mental health and socio-affective factors	Feelings of anxiety/nervousness lately	232	234		0.001	0.001	
Mental health and socio-affective factors	Feelings of inadequacy lately	233	242		0.001	0.000	

Body injuries and illnesses	Other serious medical conditions and disabilities	234	245	192	0.001	0.000	0.001
Mental health and socio-affective factors	Contemplated self-harm	235	213		0.001	0.001	
Mental health and socio-affective factors	Activities Avoided Due to Recent Stress	236	231		0.001	0.001	
Lifestyle and environment	Facial ageing	237	194	182	0.001	0.002	0.002
Mental health and socio-affective factors	Serious Life-Threatening Accident	238	220		0.001	0.001	
Mental health and socio-affective factors	Poor appetite and overeating lately	239	237		0.001	0.000	
Diet and alcohol	Injury Caused by alcohol consumption	240	240		0.001	0.000	
Mental health and socio-affective factors	Victim of sexual assault	241	218		0.0009	0.001	
Lifestyle and environment	Loud music exposure frequency	242	222	197	0.0009	0.001	0.001
Mental health and socio-affective factors	Sexually molested as a child	243	241		0.0007	0.000	
Mental health and socio-affective factors	Diminished pleasure in activities lately	244	228		0.0006	0.001	
Body injuries and illnesses	Chest pain or discomfort	245	246	196	0.0006	0.0005	0.001
Mental health and socio-affective factors	Feelings of restlessness lately	246	244		0.0006	0.0005	
Mental health and socio-affective factors	Long-lasting sadness or depression	247	250		0.0006	0.0003	
Mental health and socio-affective factors	Feelings of foreboding lately	248	247		0.0006	0.0004	
Mental health and socio-affective factors	Period of mania / excitability	249	238		0.0005	0.0008	
Early life factors	Handedness (chirality/laterality)	250	248	199	0.0003	0.0004	0.0009
Mental health and socio-affective factors	Non-consensual sexual Interference by ex-/partners	251	253		0.0002	0.0	
Mental health and socio-affective factors	Ever self-harmed	252	251		0.0001	0.0002	
Mental health and socio-affective factors	Changes in speed/amount of moving or speaking lately	253	257		0.0001	0.0	
Mental health and socio-affective factors	Having thoughts of self-harm lately	254	254		0.0001	0.0	
Mental health and socio-affective factors	Belief in a personal conspiracy	255	255		0.0	0.0	
Mental health and socio-affective factors	Belief in unreal communications	256	259		0.0	0.0	
Mental health and socio-affective factors	heard an un-real voice	257	256		0.0	0.0	
Mental health and socio-affective factors	Combat/War-Zone Involvement	258	258		0.0	0.0	
Mental health and socio-affective factors	seen an un-real vision	259	252		0.0	0.0	
Body injuries and illnesses	Cancer : diagnosis	260	243	201	0.0	0.0005	0.0001

Sociodemographics	Race	261	249	200	0.0	0.0003	0.0001

Supplementary Table 8. Summary statistics of coffee intake: Summary statistics of coffee intake (Data Field ID = 1498) in main subset (*n* = 3706).

Coffee Intake*	Cup of coffee/day**	Mean of Brain Age Gap	Count
None	0	-0.19	832
Low	1-2	-0.02	825
Moderate	2-5	0.64	612
High	5-10	0.91	493
Very High	<10	0.93	469

*: mean = 2.02 cups per day, std = 1.96, median =2, mode= 1, range= 0-20

**: The coffee intake was categorized based on the amount consumed cup of coffee per day.

Supplementary Table 9. **Summary of cereal type:** Summary of cereal type (Data Field ID = 1468) in main subset (n = 3706).

Cereal Type	Count	Percentage
Oat cereal (e.g. Ready Brek, porridge)	832	22.45
Muesli	825	22.26
Bran cereal (e.g. All Bran, Branflakes)	612	16.51
Biscuit cereal (e.g. Weetabix)	493	13.3
Other (e.g. Cornflakes, Frosties)	469	12.65
Missing values	475	12.8

Supplementary Table 10. **Summary statistics of hip circumference:** Summary statistics of hip circumference (Data Field ID = 49) in the main subset (n = 3706).

Mean (cm)	Std (cm)	Median (cm)	Mode (cm)	Range (cm)
102.00	7.99	101	100	77-168

Supplementary Table 11. Correlation Between Chronological Age and Brain Age Gap (BAG) Before and After Bias Correction.

	MAIN	REPLICATION	VARIABLES-RESTRICTED
CORRELATION (R) BEFORE CORRECTION	-0. 672	-0.674	-0.668
CORRELATION (R) AFTER CORRECTION	-0.130	-0.135	-0.123
SCATTER PLOT (CHRONOLOGICAL AGE VS. BAG)	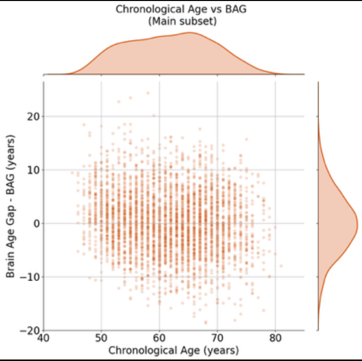	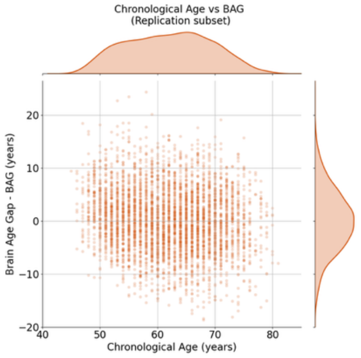	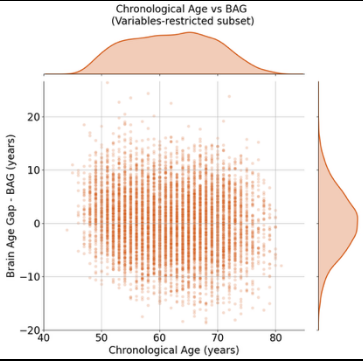

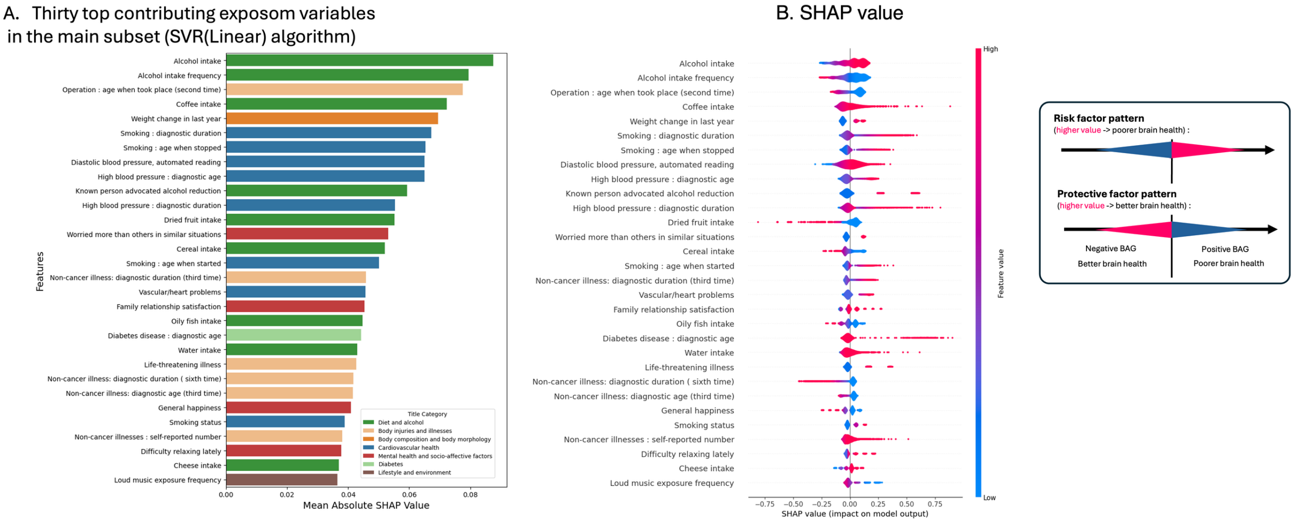

Supplementary Figure 1. **SHAP-Based Contribution of Exposome Factors to Grey Matter Health Prediction in the Main Subset Using Linear SVR:** Exposome factors' contribution to the grey matter health prediction in main subset (*n* = 3706, 261 exposome variables) using SVR (linear) algorithm. Panel A depicts mean absolute SHAP values. Panel B depicts SHAP's distributions. In this figure, SHAP stands for SHapley Additive exPlanations. Source data are provided as a Source Data file for panel A.

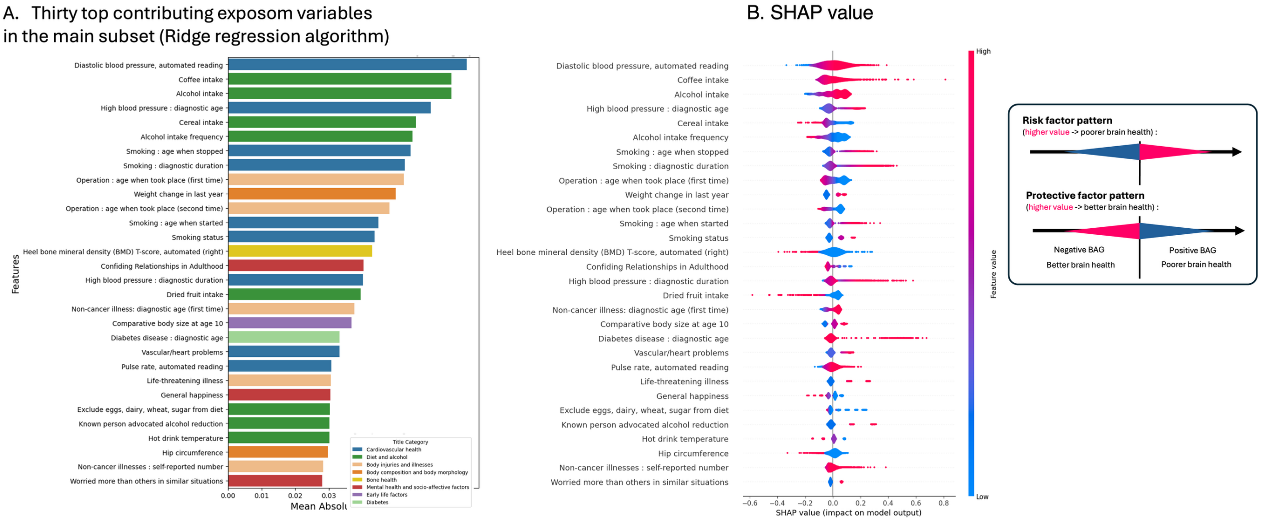

Supplementary Figure 2. **SHAP-Based Contribution of Exposome Factors to Grey Matter Health Prediction in the Main Subset Using Ridge**: Exposome factors' contribution to the grey matter health prediction in main subset (*n* = 3706, 261 exposome variables) using Ridge regression algorithm. Panel A depicts mean absolute SHAP values. Panel B depicts SHAP's distributions. In this figure, SHAP stands for SHapley Additive exPlanations. Source data are provided as a Source Data file for panel A.

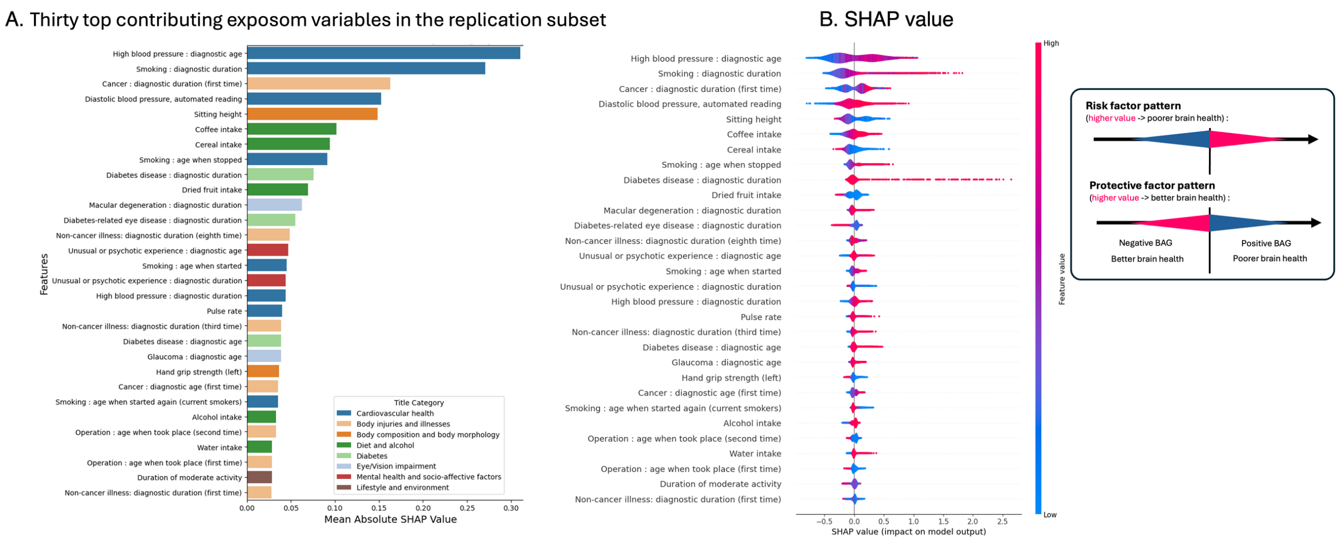

Supplementary Figure 3. SHAP-Based Contribution of Exposome Factors to Grey Matter Health Prediction in the Replication Subset Using Random Forest algorithm: Exposome factors' contribution to the grey matter health prediction in replication subset (*n* = 4202, 259 exposome variables). Panel A depicts mean absolute SHAP values. Panel B depicts SHAP's distributions. In this figure, SHAP stands for SHapley Additive exPlanations. Source data are provided as a Source Data file for panel A.

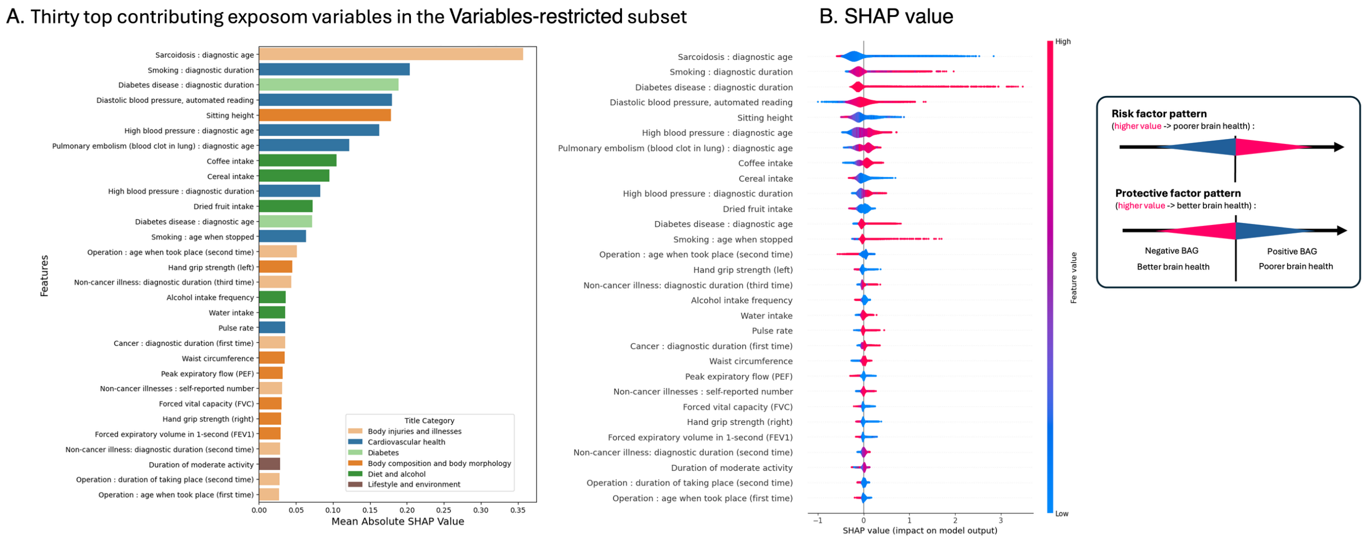

Supplementary Figure 4. SHAP-Based Contribution of Exposome Factors to Grey Matter Health Prediction in the Variables-Restricted Subset Using Random Forest algorithm: Exposome factors' contribution to the grey matter health prediction in variables-restricted subset (*n* = 7736, 201 exposome variables). Panel A depicts mean absolute SHAP values. Panel B depicts SHAP's distributions. In this figure, SHAP stands for SHapley Additive exPlanations. Source data are provided as a Source Data file for panel A.

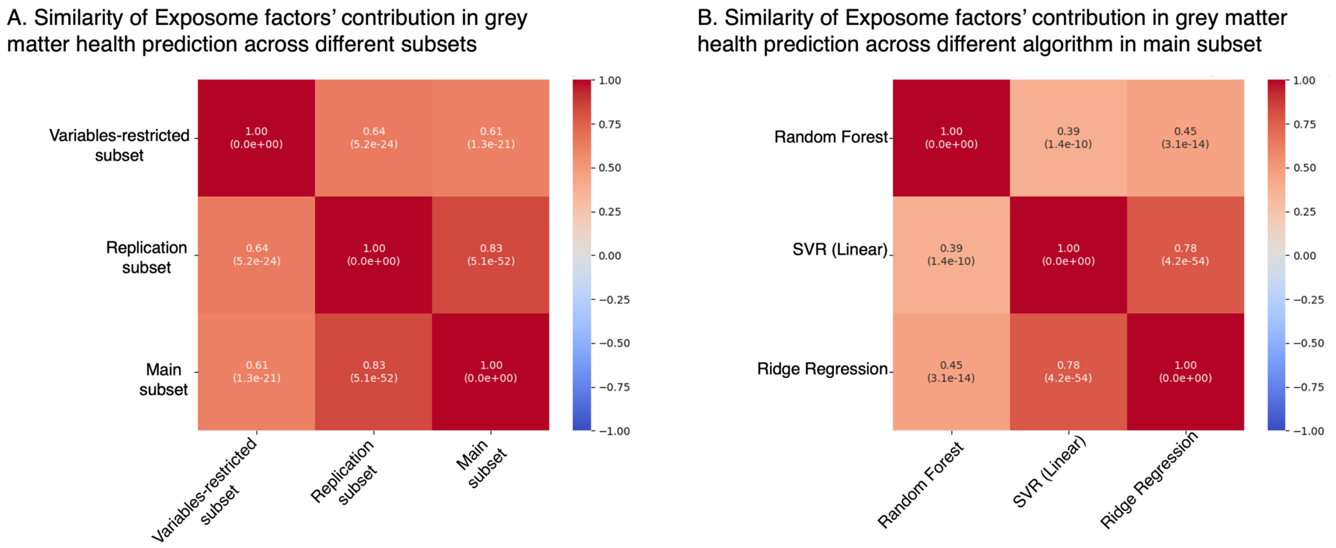

Supplementary Figure 5. **Similarity of Exposome factors' contribution in grey matter health prediction across different subsets/algorithms:** including main subset (*n* = 3706), replication subset (*n* = 4202), and variable-restricted subset (*n* = 7736). The similarity is depicted across different subsets (Panel A) and across different algorithms in the main subset (Panel B).

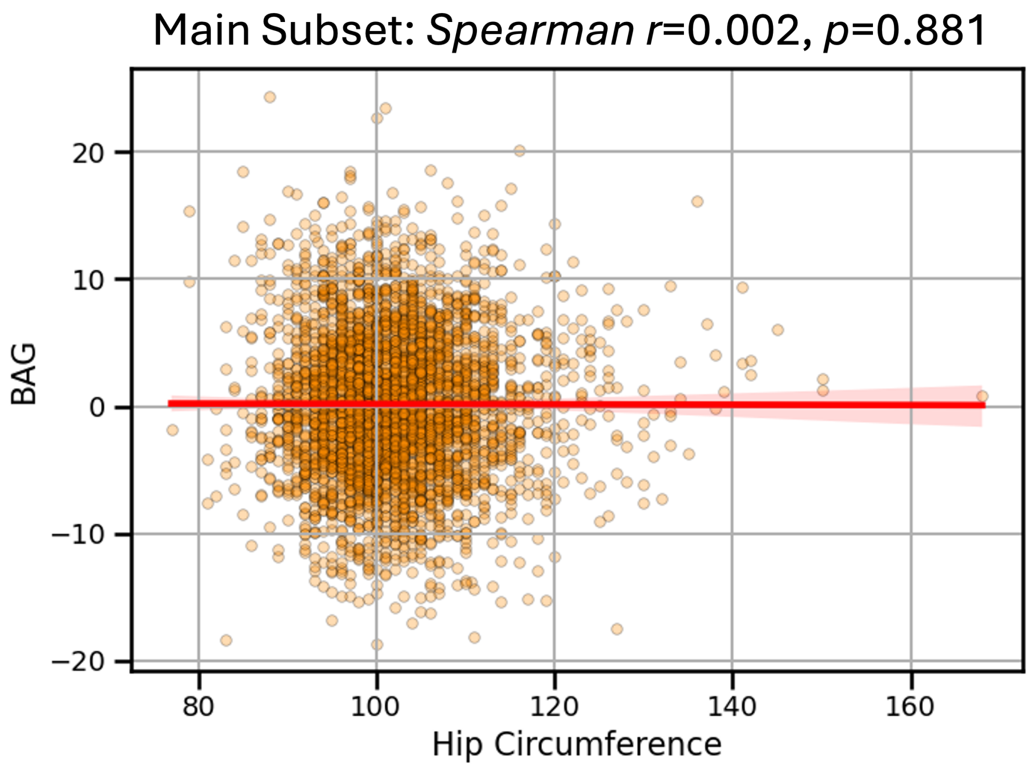

Supplementary Figure 6. **Association Between Hip Circumference and Grey Matter Health in the Main Subset:** Correlation coefficient (effect size) for association between Hip Circumference and BAG in main subset (*n* = 3706). Correlation coefficient calculated by using *Spearman r*.
In this figure, BAG stands for Brain Age Gap.

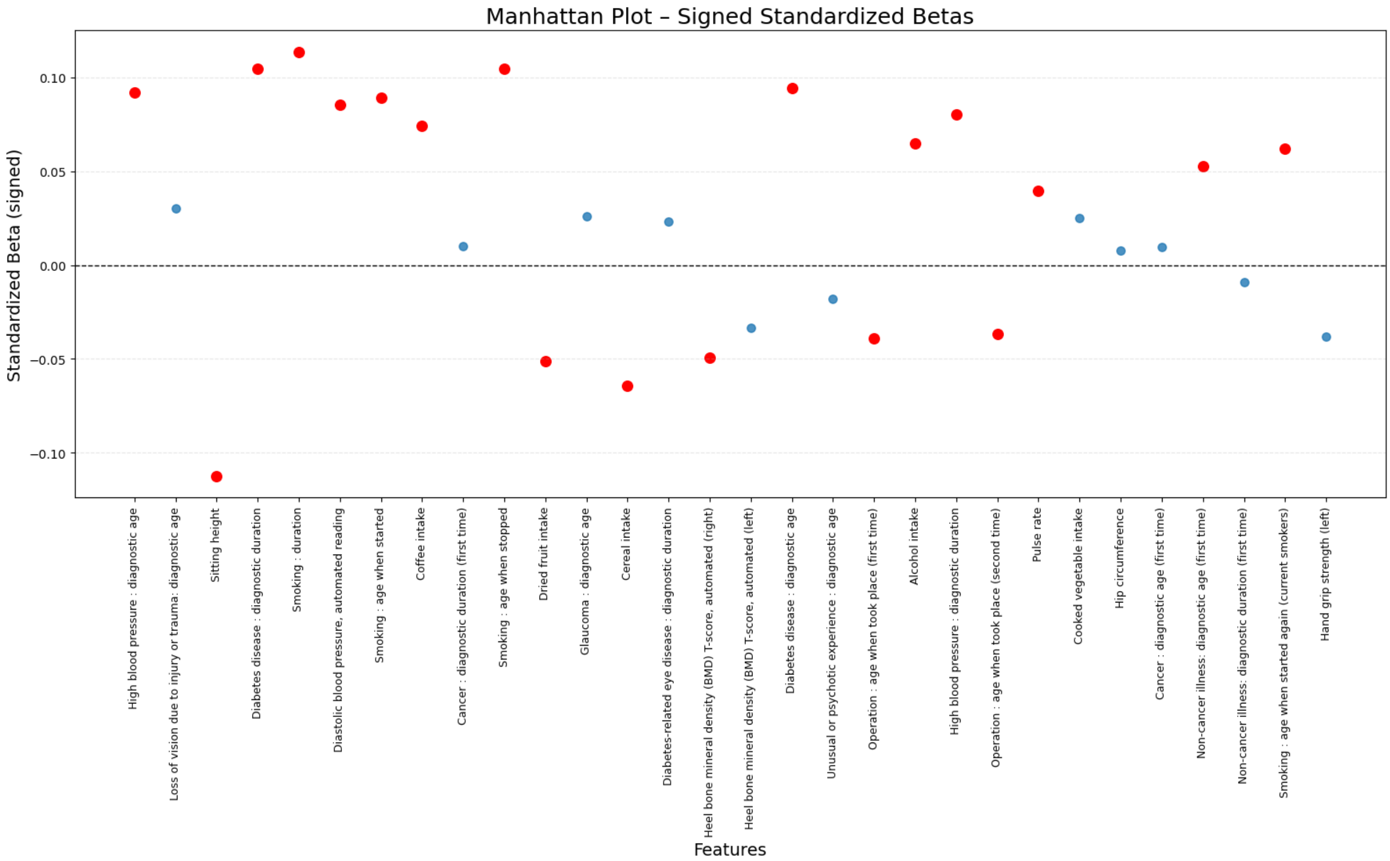

Supplementary Figure 7. **Standardized Regression Coefficients for the Top Exposome Features Associated with Grey Matter Health in the Main Subset:** Standardized regression coefficients for the top 30 exposome features associated with brain age gap (BAG) in the main subset. The figure shows standardized beta coefficients derived from generalized linear models relating each exposome feature separately to BAG, when accounting for age, age², sex, height, and volumetric scaling from T1 head image to standard space. Both predictors and outcome were standardized to allow comparison of effect magnitudes across variables. Red dots illustrate significant associations after correcting for multiple comparisons using FDR-correction (while blue dots are not significant). These analyses were conducted for reader's information about the magnitude of each association when each exposome variable is considered in isolation in relation to BAG. Source data are provided as a Source Data file.

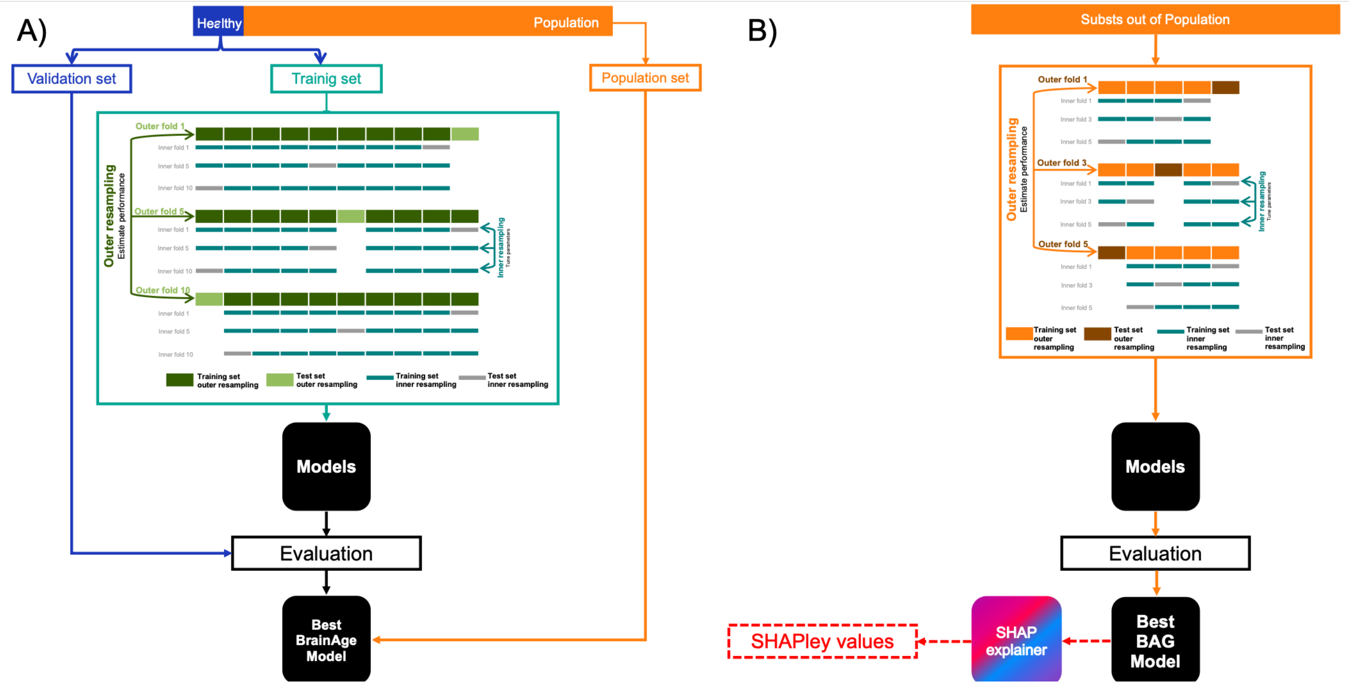

Supplementary Figure 8. **Flowchart of Model Development for Brain Age and Grey Matter Health (Brain Age Gap) Predictions:** Study design for models' development and test preventing data leakage between models, as well as between training and test sets. A) Brain Age Model development, B) Exposome-BAG-Model (Brain Health Model).

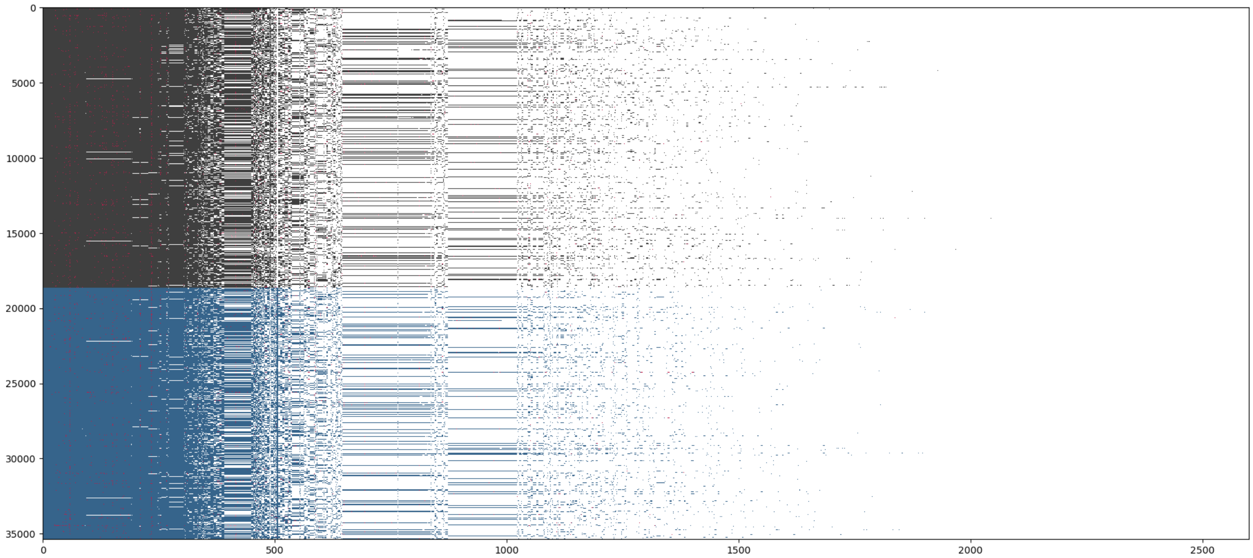

Supplementary Figure 9. **Missingness pattern in the initial list of selected exposome variables:** Missingness pattern in the literature-based a-priori selected data fields (Missingno package). The grey colours are the filled values in women participants. Blue colours are the filled values in men participants. White colour reflects missing values, and red colour indicates non-informative answers (i.e., I don't know, I prefer not to answer etc,.).

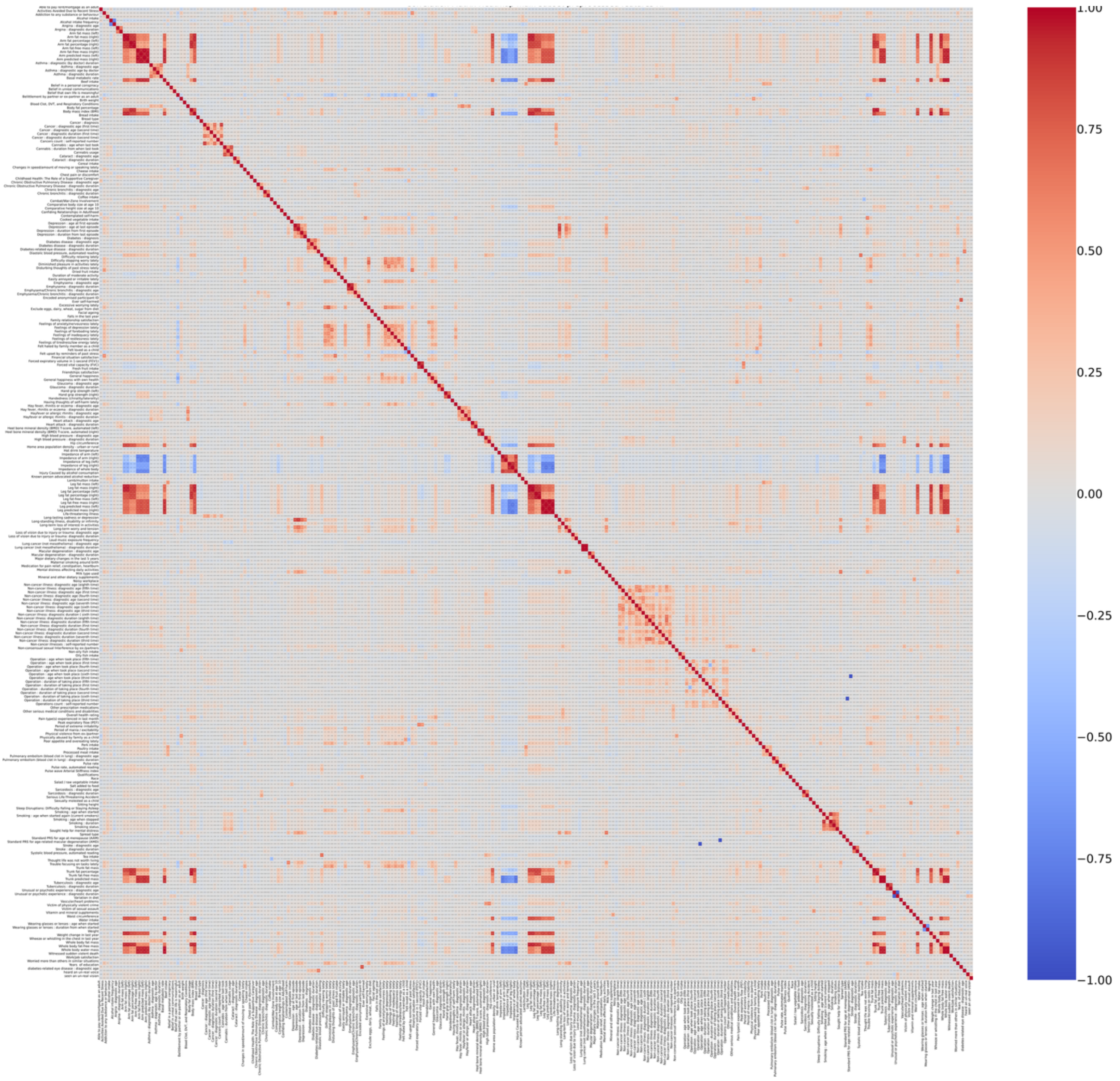

Supplementary Figure 10. **correlation heatmap of the preprocessed features:** Clusters of more correlated variables pertain to body composition and body morphology variables.

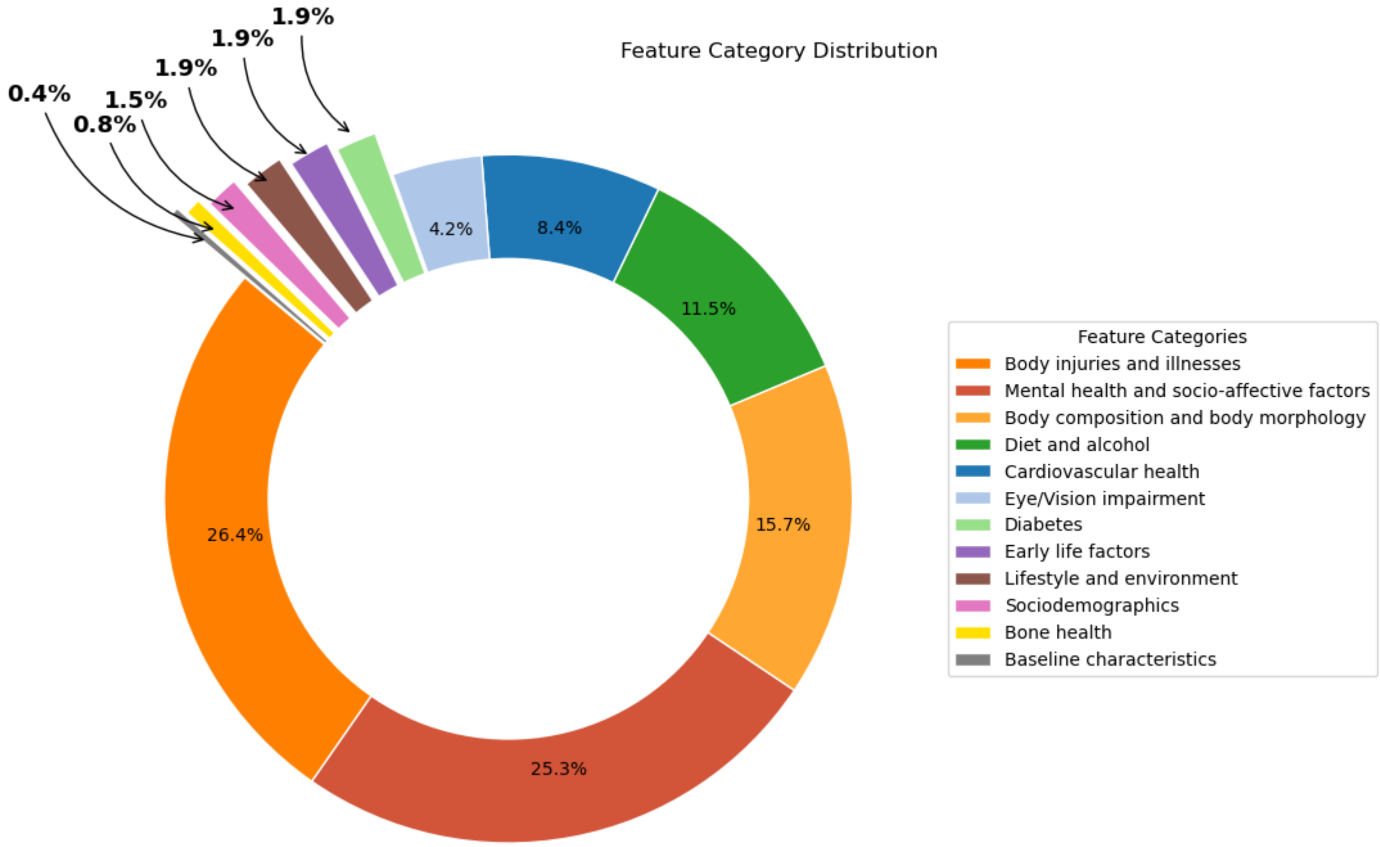

Supplementary Figure 11. Distribution of exposome variables across different exposome domains: Source data are provided as a Source Data file.

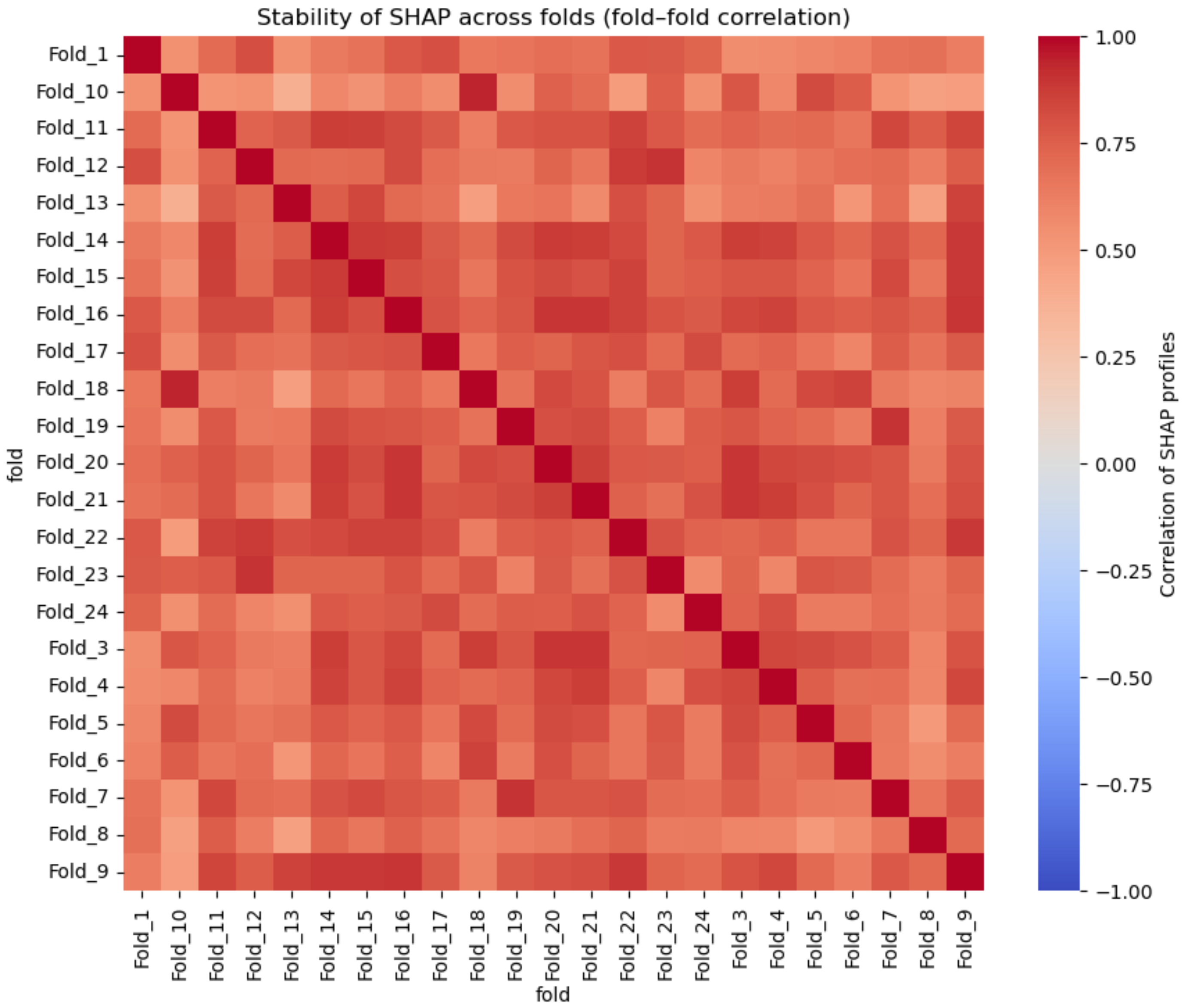

Supplementary Figure 12. Fold-to-fold mean absolute SHAP values' correlations: In this figure, SHAP stands for SHapley Additive exPlanations.
