# Supplementary material for: Exposome-wide patterns predict brain health in aging
Source: Nat Commun. 2026 Apr 10;17:3409. doi: 10.1038/s41467-026-71271-9 (PMC13068930; doi:10.1038/s41467-026-71271-9)
Supplement: Supplementary file 2 — Reporting Summary [file 41467_2026_71271_MOESM2_ESM.pdf]

nature portfolio

Corresponding author(s): Mostafa Mahdipour & Sarah Genon

Last updated by author(s): Jan 26, 2026

Reporting Summary

Nature Portfolio wishes to improve the reproducibility of the work that we publish. This form provides structure for consistency and transparency in reporting. For further information on Nature Portfolio policies, see our Editorial Policies and the Editorial Policy Checklist.

Statistics

For all statistical analyses, confirm that the following items are present in the figure legend, table legend, main text, or Methods section.

n/a	Confirmed	
☐	☒	The exact sample size (*n*) for each experimental group/condition, given as a discrete number and unit of measurement
☐	☒	A statement on whether measurements were taken from distinct samples or whether the same sample was measured repeatedly
☐	☒	The statistical test(s) used AND whether they are one- or two-sided<br>*Only common tests should be described solely by name; describe more complex techniques in the Methods section.*
☐	☒	A description of all covariates tested
☐	☒	A description of any assumptions or corrections, such as tests of normality and adjustment for multiple comparisons
☐	☒	A full description of the statistical parameters including central tendency (e.g. means) or other basic estimates (e.g. regression coefficient) AND variation (e.g. standard deviation) or associated estimates of uncertainty (e.g. confidence intervals)
☐	☒	For null hypothesis testing, the test statistic (e.g. *F*, *t*, *r*) with confidence intervals, effect sizes, degrees of freedom and *P* value noted<br>*Give P values as exact values whenever suitable.*
☒	☐	For Bayesian analysis, information on the choice of priors and Markov chain Monte Carlo settings
☒	☐	For hierarchical and complex designs, identification of the appropriate level for tests and full reporting of outcomes
☐	☒	Estimates of effect sizes (e.g. Cohen's *d*, Pearson's *r*), indicating how they were calculated

Our web collection on statistics for biologists contains articles on many of the points above.

Software and code

Policy information about availability of computer code

Data collection	Detail explanation of data collection in UK Biobank cohort can be found in https://www.ukbiobank.ac.uk/media/gnkeyh2q/study-rationale.pdf.
Data analysis	The analysis code is publicly available on GitHub: https://github.com/MostafaMahdipour/Predicting_Brain_Age_Gap_BAG_using_UKB_exposome

For manuscripts utilizing custom algorithms or software that are central to the research but not yet described in published literature, software must be made available to editors and reviewers. We strongly encourage code deposition in a community repository (e.g. GitHub). See the Nature Portfolio guidelines for submitting code & software for further information.

Data

Policy information about availability of data

All manuscripts must include a data availability statement. This statement should provide the following information, where applicable:

- Accession codes, unique identifiers, or web links for publicly available datasets
- A description of any restrictions on data availability
- For clinical datasets or third party data, please ensure that the statement adheres to our policy

The brain imaging and exposome data used in this study are derived from the UK Biobank resource under application number 41655. These data are available under restricted access due to ethical approval requirements, participant consent, and data protection regulations. Raw participant-level data are protected and cannot be publicly shared due to data privacy laws. Access can be obtained by bona fide researchers through application to the UK Biobank Access Management System (https://www.ukbiobank.ac.uk/), subject to approval by UK Biobank. Processed data that do not contain participant-level information are provided with this paper

as Source Data files, where applicable.

Research involving human participants, their data, or biological material

Policy information about studies with human participants or human data. See also policy information about sex, gender (identity/presentation), and sexual orientation and race, ethnicity and racism.

Reporting on sex and gender	The term 'sex' in this study refers to a biological attribute. Sex information was obtained through self-report. Predictive models for age and brain grey matter health were developed using data from all participants, regardless of sex. Where applicable, sex was included as a covariate in the predictive models.
Reporting on race, ethnicity, or other socially relevant groupings	Ethnic background of participants in UK Biobank were available
Population characteristics	This study focus on participants with available imaging data (39390 participants, aged 44-82 years, mean 63.64 ± 7.54 years, n = 20707 females). Cognitively healthy participants ("'healthy sample") had no self-reported long-standing illness disability or infirmity (UK Biobank data field #2188), no self-reported diabetes (field #2443), no stroke history (field #4056), no ICD-10 diagnosis and good or excellent self-reported health (field #2178). These criteria were defined in line with a previous study11 and led to a sample of 5025 healthy participants (age range 46-82 years, mean 62.12 years ± std 7.16 years, 2579 females), while leaving 34365 participants (age range: 44-82 years, mean 63.86 ±7.57 years, 18128 females) to define subsets for exposome based prediction of grey matter health (see Supplementary Table 2).
Recruitment	Participants were recruited by the UK Biobank.
Ethics oversight	The UK Biobank has approval from the North West Multi-center Research Ethics Committee (MREC) (https://www.ukbiobank.ac.uk/learn-more-about-uk-biobank/about-us/ethics) to obtain and disseminate data and sample from the participants Written informede consent was obtained from all participants.

Note that full information on the approval of the study protocol must also be provided in the manuscript.

Field-specific reporting

Please select the one below that is the best fit for your research. If you are not sure, read the appropriate sections before making your selection.

[x] Life sciences [ ] Behavioural & social sciences [ ] Ecological, evolutionary & environmental sciences

For a reference copy of the document with all sections, see nature.com/documents/nr-reporting-summary-flat.pdf

Life sciences study design

All studies must disclose on these points even when the disclosure is negative.

Sample size	Sample size was determined by the data availability from the UK Biobank.
Data exclusions	Participants with missing responses in either the T1-weighted MRI imaging data or any of the exposome variables were excluded.
Replication	To verify the reproducibility of our findings, we conducted analyses across three partially overlapping participant subsets, each offering a different balance between sample size and exposome variable coverage. First, we defined a main analysis subset consisting of 3,706 participants who had complete data for 261 exposome variables. To assess replicability, we constructed a replication subset by removing two variables (left and right heel bone density), allowing the inclusion of a larger sample of 4,292 participants with otherwise comparable exposome coverage. Finally, we identified a variables-restricted subset of 7,736 participants with data available for 201 exposome variables. This subset excluded primarily socio-affective and mental health-related variables, enabling us to test the robustness of our predictive models across a broader participant base with reduced variable coverage.
Randomization	Participants were not assigned to experimental groups; therefore, randomization was not applicable. Additional details regarding the experimental design can be found in Sudlow et al., PLOS Medicine (2013).
Blinding	Participants were not assigned to experimental groups during data collection, data processing/or statistical analyses. Blinding was therefore not applicable.

Reporting for specific materials, systems and methods

We require information from authors about some types of materials, experimental systems and methods used in many studies. Here, indicate whether each material, system or method listed is relevant to your study. If you are not sure if a list item applies to your research, read the appropriate section before selecting a response.

Materials & experimental systems

n/a	Involved in the study
☒	☐ Antibodies
☒	☐ Eukaryotic cell lines
☒	☐ Palaeontology and archaeology
☒	☐ Animals and other organisms
☒	☐ Clinical data
☒	☐ Dual use research of concern
☒	☐ Plants

Methods

n/a	Involved in the study
☒	☐ ChIP-seq
☒	☐ Flow cytometry
☐	☒ MRI-based neuroimaging

Plants

Seed stocks

Report on the source of all seed stocks or other plant material used. If applicable, state the seed stock centre and catalogue number. If plant specimens were collected from the field, describe the collection location, date and sampling procedures.

Novel plant genotypes

Describe the methods by which all novel plant genotypes were produced. This includes those generated by transgenic approaches, gene editing, chemical/radiation-based mutagenesis and hybridization. For transgenic lines, describe the transformation method, the number of independent lines analyzed and the generation upon which experiments were performed. For gene-edited lines, describe the editor used, the endogenous sequence targeted for editing, the targeting guide RNA sequence (if applicable) and how the editor was applied.

Authentication

Describe any authentication procedures for each seed stock used or novel genotype generated. Describe any experiments used to assess the effect of a mutation and, where applicable, how potential secondary effects (e.g. second site T-DNA insertions, mosiacism, off-target gene editing) were examined.

Magnetic resonance imaging

Experimental design

Design type

Structural MRI

Design specifications

No specific experimental setup used in this study, not applicable.

Behavioral performance measures

No behavioral performance conducted during the scan, not applicable.

Acquisition

Imaging type(s)

T1 weighted MRI images

Field strength

3 Tesla

Sequence & imaging parameters

Imagine aquisition details were described by Miller et al. Nature medicine (2016).

Area of acquisition

whole brain

Diffusion MRI ☐ Used ☐ Not used

Preprocessing

Preprocessing software

For preprocessing of the T1-weighted MRI imaging data, we used an in-house developed framework designed for computationally reproducible processing of large-scale data (FAIRly big). Specifically, a Singularity container was created that included a pipeline to perform voxel-based morphometry (VBM) on individual T1-weighted MRI images using the Computational Anatomy Toolbox (CAT). All T1-weighted anatomical scans were processed using CAT version 12.7.

Normalization

Normalization was done by CAT12 (Version r1720)(https://neuro-jena.github.io/software.html#cat) with default settings compiled under Matlab 2019b.

Normalization template

MNI ICBM 152 nonlinear

Noise and artifact removal

It is not applicable for structural MRI.

Volume censoring

No volume censoring performed on this data.

Statistical modeling & inference

Model type and settings

Predictive(Linear Regression, Ridge Regression, Support Vector Regression (SVR) and Random Forest)

Effect(s) tested

No task or stimulation conditions involved in this study.

Specify type of analysis: ☐ Whole brain ☐ ROI-based ☒ Both

Anatomical location(s): Whole brain grey matter was parcellated using a combination of the Schaefer atlas for 200, 400, 600, 800 and 1000 cortical regions and the Melbourne subcortex atlas for 32 and 54 subcortical regions leading to five levels of representations of grey matter (grey matter volume for either 232, 454, 654, 854 and 1054 regions).

Statistic type for inference (See Eklund et al. 2016): No whole brain voxel-wised or cluster-based analyses involved in this study and thus not applicable.

Correction: The false discovery rate (FDR) was controlled at 5% using Benjamini-Hochberg procedure.

Models & analysis

n/a	Involved in the study
☒	☐ Functional and/or effective connectivity
☒	☐ Graph analysis
☐	☒ Multivariate modeling or predictive analysis

Multivariate modeling and predictive analysis: Four predictive algorithms including Linear Regression, Ridge Regression, Support Vector Regression (SVR), and Random Forest were evaluated to design a Brain Age Prediction Model. They were all trained to predict an individual's chronological age using the five different sets of grey matter features (with different levels of granularity) in healthy sample (n=5025). Predictive brain age models were trained on 80% of the healthy sample (n = 4020) using 10-fold nested cross-validation with 5 repeats for estimating the chronological age based on the five different sets of individual's grey matter features. Furthermore, a 10-folds inner cross-validation loop was implemented for hyperparameter tuning using a grid search approach. The optimized brain age models were then validated on held-out set made by the remaining 20% of the healthy sample (n = 1005). Finally, the model with the minimum prediction error in held-out set was selected and fitted on the entire healthy sample (training + held-out) and used to estimate the chronological age in population set (n=34365, age range: 44-82 years, mean 63.86 ±7.57 years, 18128 females) in the UK Biobank dataset.

To predict grey matter health at the individual level based on a set of exposome variables, we first implemented a random forest algorithm which used a decision tree-based approach and has the advantage of accounting for non-linear relationships between the exposome variables and grey matter health. Random Forest was trained and tested for each subset (i.e. the main subset (n=3706, 261distinct measure of exposome variables), the replication subset (n=4292, 259 distinct measure of exposome variables), and the "'variables-restricted"' subset (n=7736, 201distinc measure of exposome variables), separately), However, for the sake of replication, we also implemented two additional popular algorithms: ridge regression (which was also used for the brain age model) and Support Vector Regression (SVR).
